# Supplementary material for: Promiscuous Targeting of Cellular Proteins by Vpr Drives Systems-Level Proteomic Remodeling in HIV-1 Infection
Source: Cell Rep. 2019 Apr 30;27(5):1579–1596.e7. doi: 10.1016/j.celrep.2019.04.025 (PMC6506760; doi:10.1016/j.celrep.2019.04.025)
Supplement: Document S1. Figures S1 and S2 and Table S2 [file mmc1.pdf]

Cell Reports, Volume *27*

Supplemental Information

Promiscuous Targeting of Cellular Proteins

by Vpr Drives Systems-Level Proteomic Remodeling

in HIV-1 Infection

Edward J.D. Greenwood, James C. Williamson, Agata Sienkiewicz, Adi Naamati, Nicholas J. Matheson, and Paul J. Lehner

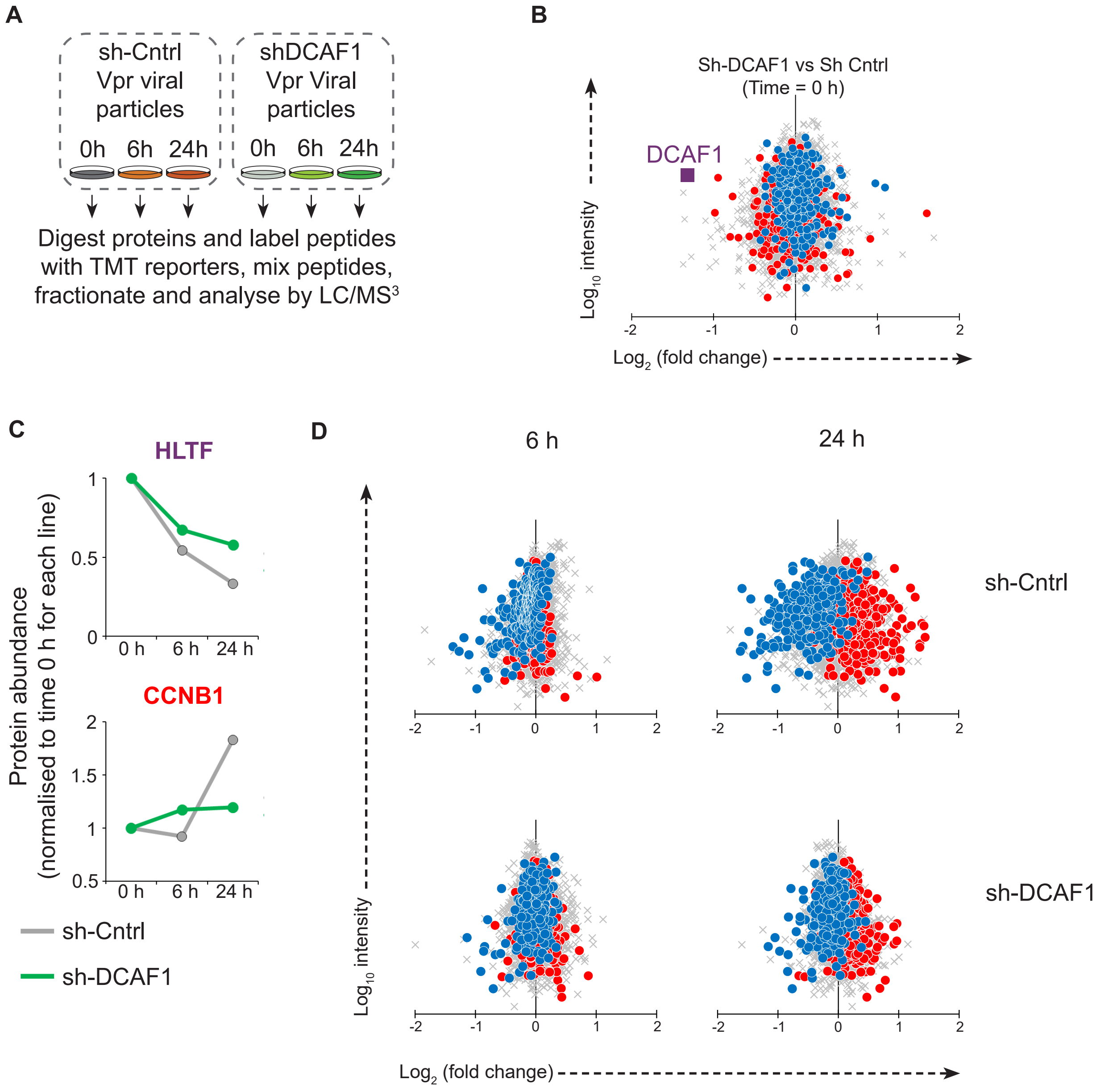

Figure S1. Quantifying the effect of Vpr under reduced DCAF1 conditions. Related to Figure 2.
A, Graphical summary of the DCAF1 KD experiment. **B**, Scatterplot displaying pairwise comparison between Sh-Control and Sh-DCAF1 cells at 0 h, with defined groups of 302 Vpr depleted (blue) and 413 increased (red) proteins highlighted. DCAF1 is highlighted in purple. **C**, Example time-course behaviour for one Vpr target (HLTF) and one secondary Vpr effect (CCNB1). **D**, Scatterplots showing the pairwise comparison between the 6 or 24 h time-point with the 0 h condition for each sh-transduced cell line.

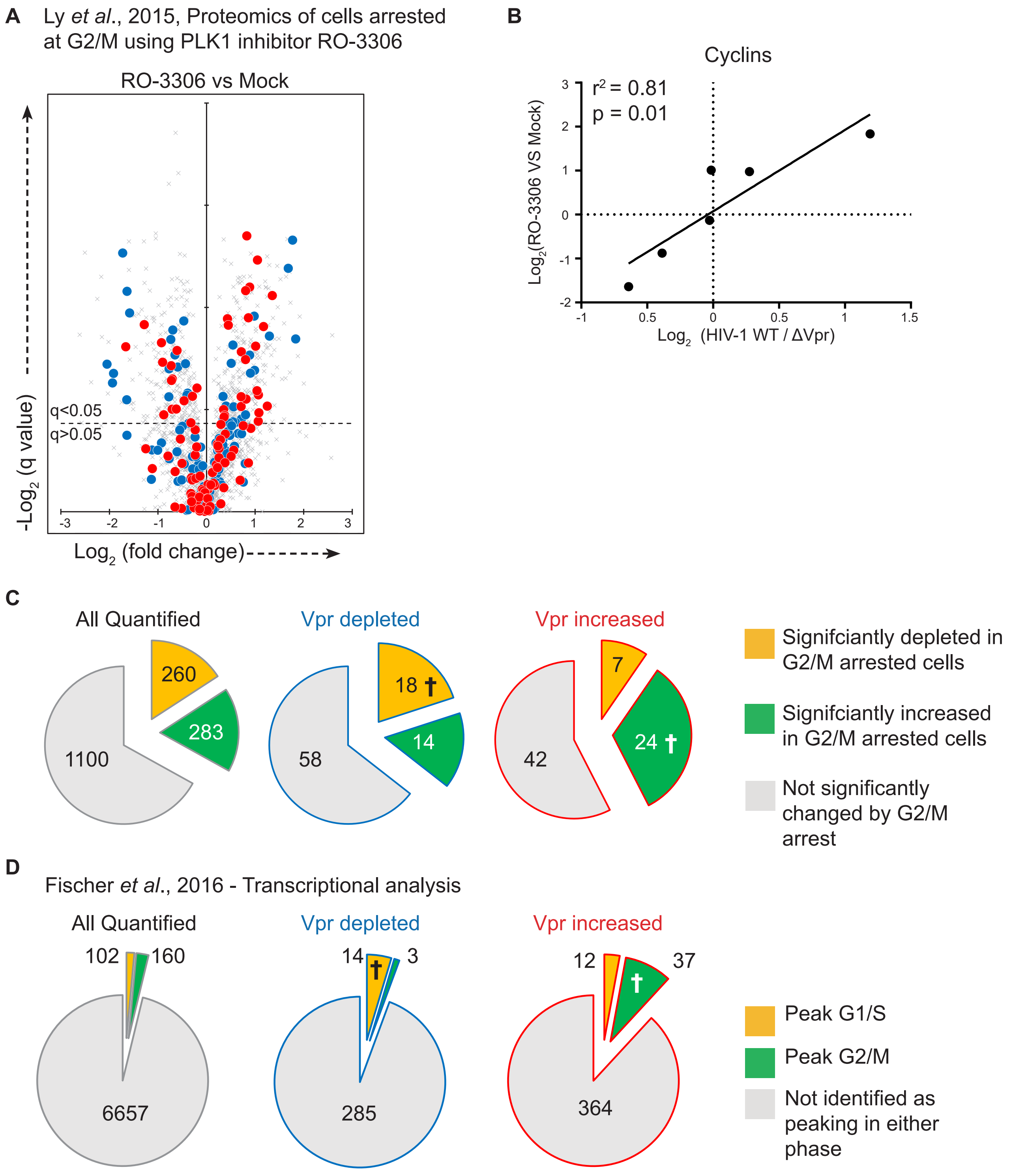
A
Ly et al., 2015, Proteomics of cells arrested at G2/M using PLK1 inhibitor RO-3306
RO-3306 vs Mock
q<0.05
q>0.05
-Log2 (q value)
Log2 (fold change)
-3 -2 -1 0 1 2 3
B
Cyclins
r2 = 0.81
p = 0.01
Log2(RO-3306 VS Mock)
Log2 (HIV-1 WT / ΔVpr)
3 2 1 0 -1 -2
-1 0.5 0 0.5 1 1.5
C
All Quantified
260
283
1100
Vpr depleted
18 †
14
58
Vpr increased
7
24 †
42
Signifciantly depleted in G2/M arrested cells
Signifciantly increased in G2/M arrested cells
Not significantly changed by G2/M arrest
D
Fischer et al., 2016 - Transcriptional analysis
All Quantified
102
160
6657
Vpr depleted
14
3
†
285
Vpr increased
12
37
†
364
Peak G1/S
Peak G2/M
Not identified as peaking in either phase

Figure S2. Cell cycle regulation of Vpr modulated proteins. Related to Figure 2.
A, Ly *et al*., 2015 contains a proteomic analysis of NB4 cells arrested in G2/M with the PLK1 inhibtor RO-3306. Scatterplot showing the pairwise comparison of abundance of proteins isolated from RO-3306 treated vs mock treated cells. Groups of proteins defined in the current study of 302 Vpr depleted (blue) and 413 increased (red) proteins are highlighted, indicating the behaviour of these proteins in NB4 cells arrested at G2/M. **B**, Correlation of the Vpr mediated change in cyclin abundance in the present study (x-axis), with RO-3306 mediated changes in NB4 cells in Ly *et al*., 2015. Changes in cyclin abundance in HIV infection are assumed to be secondary to cell cycle arrest, and thus this correlation indicates the concordance between effects secondary to cell cycle arrest between the two datasets. **C**, Pie charts showing the overlap between changes in the present study and changes induced by G2/M arrest in Ly *et al*., 2015. Left panel shows the behaviour of all proteins quantified in both the present study (Figure 1A and Figure 2A) and Ly *et al*., 2015, i.e. a total of 1643 proteins were quantified in both datasets, of which 1100 proteins did not significantly change in RO-3306 G2/M arrest, 260 proteins were significantly depleted in G2/M arrested cells, and 283 proteins were significantly increased in G2/M arrested cells. Middle panel shows the behaviour the defined group of 302 Vpr depleted proteins. I.e. of 302 proteins, a total of 90 proteins were detected in Ly *et al*., 2015, 58 of which did not significantly change in G2/M arrest, 18 were significantly depleted in G2/M arrest and 14 were significantly increased in G2/M arrest. Right panel shows the behaviour of the defined group of 413 Vpr increased proteins. † Indicates the fraction in each case where the change induced by cell cycle arrest is in the same direction as the effect of Vpr, i.e. the fraction for which cell cycle arrest could explain the Vpr mediated protein changes. **D**, Fischer *et al*., 2016, use a meta-analysis of transcriptional datasets to define lists of 115 proteins whose expression peaks in G1/S phase and 174 proteins whose expression peaks in G2/M phase. Pie-charts show the overlap between these lists and (left) all proteins detected in the present study, (middle) proteins defined as being depleted by Vpr, and (right) proteins defined as being increased by Vpr. As in the proteomics dataset, there is some enrichment of proteins with peak expression in G2/M within Vpr increased proteins, and some enrichment of proteins with peak expression in G1/S in Vpr depleted proteins, consistent with some effects being secondary to cell cycle arrest, but these effects are in the minority.

Table S2. Behaviour of detected previously described Vpr target proteins within these datasets. Related to Table 1.

Accession	Gene	Vpr necessary	Incoming Vpr sufficient	Degraded within 6 h	Co-IP	In direct target list (Table 1)
Q14527	HLTF	Yes	Yes	Yes	-	Yes
Q7L590	MCM10	Yes	Yes	ND[a]	-	No - protein not quantitated in in 6 hour pulsed SILAC or IP-MS
Q8N5A5	ZGPAT	Yes	Yes	Yes	-	Yes
Q96AY2	EME1	Yes	Yes	NS	-	No - depleted but with a Sig.B value of >0.01 in the pulsed SILAC experiment, not detected in IP
Q96NY9	MUS81	Yes	Yes	Yes	-	Yes
Q6N021	TET2	Yes	ND	ND	-	No - protein not quantitated in incoming Vpr experiment
P13051	UNG	Yes	Yes	NS	-	No - depleted but with a Sig.B value of >0.01 in the pulsed SILAC experiment. Detected in the IP-MS but with a single peptide

[a]ND – not detected or quantitated in this experiment.
