# Supplementary material for: Promiscuous Targeting of Cellular Proteins by Vpr Drives Systems-Level Proteomic Remodeling in HIV-1 Infection
Source: Cell Rep. 2019 Apr 30;27(5):1579–1596.e7. doi: 10.1016/j.celrep.2019.04.025 (PMC6506760; doi:10.1016/j.celrep.2019.04.025)
Supplement: Document S2. Article plus Supplemental Information [file mmc3.pdf]

Resource

Cell Reports

Promiscuous Targeting of Cellular Proteins by Vpr Drives Systems-Level Proteomic Remodeling in HIV-1 Infection

Graphical Abstract

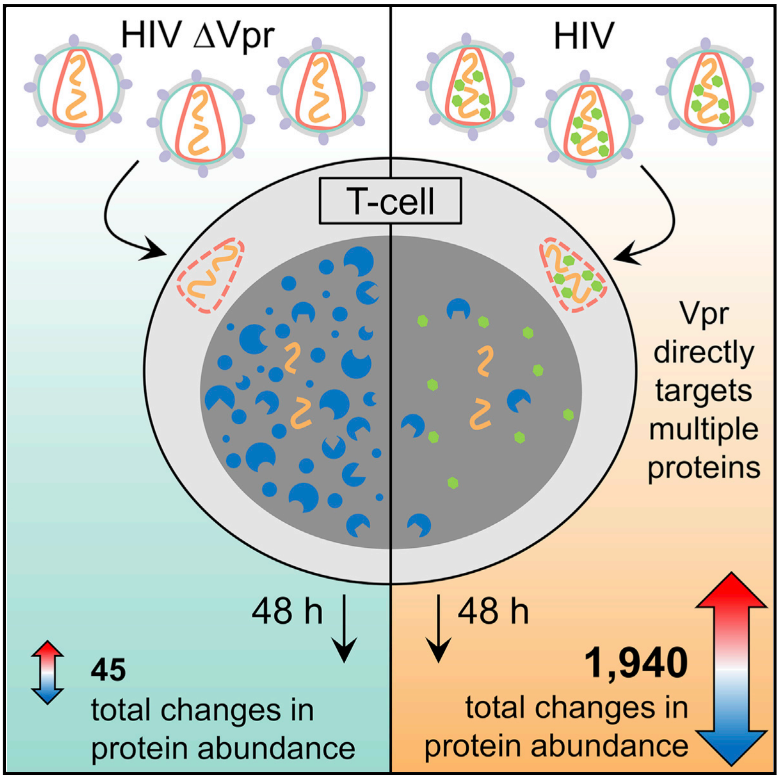

Highlights

- HIV-1 Vpr is responsible for almost all proteomic changes in HIV-1-infected cells
- Vpr directly targets multiple nuclear proteins for degradation
- Vpr cellular phenotypes (e.g., cell cycle arrest) stem from broad substrate targeting
- Targeting of a few proteins is conserved across diverse primate lentiviral species

Authors

Edward J.D. Greenwood,
James C. Williamson, Agata Sienkiewicz,
Adi Naamati, Nicholas J. Matheson,
Paul J. Lehner

Correspondence

ejdg2@cam.ac.uk (E.J.D.G.),
jcw76@cam.ac.uk (J.C.W.),
pjl30@cam.ac.uk (P.J.L.)

In Brief

HIV infection results in global changes to the cellular proteome. Greenwood et al. show that one HIV protein, Vpr, is directly or indirectly responsible for almost all of these changes by targeting multiple proteins for degradation. This broad substrate specificity explains the difficulties to date in understanding this enigmatic protein.

Greenwood et al., 2019, Cell Reports *27*, 1579–1596
April 30, 2019 © 2019 The Authors.
https://doi.org/10.1016/j.celrep.2019.04.025

OPEN ACCESS
CellPress

Promiscuous Targeting of Cellular Proteins by Vpr Drives Systems-Level Proteomic Remodeling in HIV-1 Infection

Edward J.D. Greenwood,[1,2,3,4,5,*] James C. Williamson,[1,2,3,4,*] Agata Sienkiewicz,[1,2,3] Adi Naamati,[1,3] Nicholas J. Matheson,[1,3] and Paul J. Lehner[1,2,3,*]
[1]Department of Medicine, Cambridge Biomedical Campus, University of Cambridge, Cambridge CB2 0QQ, UK
[2]Cambridge Institute for Medical Research, Keith Peters Building, Cambridge Biomedical Campus, University of Cambridge, Cambridge CB2 0XY, UK
[3]Cambridge Institute of Therapeutic Immunology and Infectious Disease, Jeffrey Cheah Biomedical Centre, Cambridge Biomedical Campus, University of Cambridge, Cambridge CB2 0AW, UK
[4]These authors contributed equally
[5]Lead Contact
*Correspondence: ejdg2@cam.ac.uk (E.J.D.G.), jcw76@cam.ac.uk (J.C.W.), pjl30@cam.ac.uk (P.J.L.)
https://doi.org/10.1016/j.celrep.2019.04.025

SUMMARY

HIV-1 encodes four "accessory proteins" (Vif, Vpr, Vpu, and Nef), dispensable for viral replication *in vitro* but essential for viral pathogenesis *in vivo*. Well characterized cellular targets have been associated with Vif, Vpu, and Nef, which counteract host restriction and promote viral replication. Conversely, although several substrates of Vpr have been described, their biological significance remains unclear. Here, we use complementary unbiased mass spectrometry-based approaches to demonstrate that Vpr is both necessary and sufficient for the DCAF1/DDB1/CUL4 E3 ubiquitin ligase-mediated degradation of at least 38 cellular proteins, causing systems-level changes to the cellular proteome. We therefore propose that promiscuous targeting of multiple host factors underpins complex Vpr-dependent cellular phenotypes and validate this in the case of G2/M cell cycle arrest. Our model explains how Vpr modulates so many cell biological processes and why the functional consequences of previously described Vpr targets, identified and studied in isolation, have proved elusive.

INTRODUCTION

The HIV-1 "accessory proteins" Vif, Vpr, Vpu, and Nef function by binding host proteins and recruiting them to cellular degradation machinery, resulting in depletion of these target substrates (Matheson et al., 2016; Simon et al., 2015; Sugden et al., 2016; Sumner et al., 2017). Some targets of Vif, Vpu, and Nef, such as APOBEC3 family members, Tetherin, and SERINC3/SERINC5, are dominantly acting viral restriction factors, and their degradation is therefore thought to directly enhance *in vivo* viral replication. Conversely, the role of Vpr in enhancing viral replication remains unclear.

Unlike other accessory proteins, Vpr is packaged into nascent viral particles and delivered into newly infected cells, and it is therefore present in the earliest stages of the viral replication cycle. Although Vpr does not enhance *in vitro* viral replication in most experimental systems, a number of cellular phenotypes have been ascribed to it; it has been described as an "enigmatic multitasker" (Guenzel et al., 2014). For example, expression of Vpr has variously been reported to cause arrest of cycling cells at the G2/M phase, apoptosis, enhancement of HIV gene expression, and stimulation or inhibition of key signaling pathways such as nuclear factor κB (NF-κB) and nuclear factor of activated T cells (NFAT) (Ayyavoo et al., 1997; Felzien et al., 1998; Lahti et al., 2003; Re et al., 1995; Roux et al., 2000).

Although the mechanisms by which Vpr causes such complex effects is controversial, most reports agree that they depend on Vpr interacting with a cellular E3 ligase complex containing DCAF1, DDB1, and Cul4 (Dehart and Planelles, 2008; Le Rouzic et al., 2007). As with the other accessory proteins, Vpr is therefore presumed to function by recruiting cellular factors to this E3 ligase complex, resulting in their subsequent degradation. Accordingly, several host factors depleted by Vpr have been identified, but their connection to Vpr-associated cell biological phenotypes is generally unclear, as is their role in regulating viral replication *in vivo* (Hofmann et al., 2017; Hrecka et al., 2016; Laguette et al., 2014; Lahouassa et al., 2016; Lv et al., 2018; Maudet et al., 2013; Romani et al., 2015; Schröfelbauer et al., 2005; Zhou et al., 2016).

We previously used unbiased quantitative proteomics to map temporal changes in cellular protein abundance during HIV infection of CEM-T4 T cells and identify targets of Vpu (SNAT1), Nef (SERINC3/5), and Vif (PPP2R5A-E) (Greenwood et al., 2016; Matheson et al., 2015). Nonetheless, known accessory protein targets only account for a tiny fraction of all HIV-dependent protein changes observed in our experiments (Greenwood et al., 2016). Given the varied cell biological phenotypes ascribed to Vpr, we hypothesized that it may be responsible for some of the remaining changes. Therefore, in this study, we undertake a comprehensive analysis of the effects of Vpr on

This is an open access article under the CC BY license (http://creativecommons.org/licenses/by/4.0/).

OPEN
ACCESS
CellPress

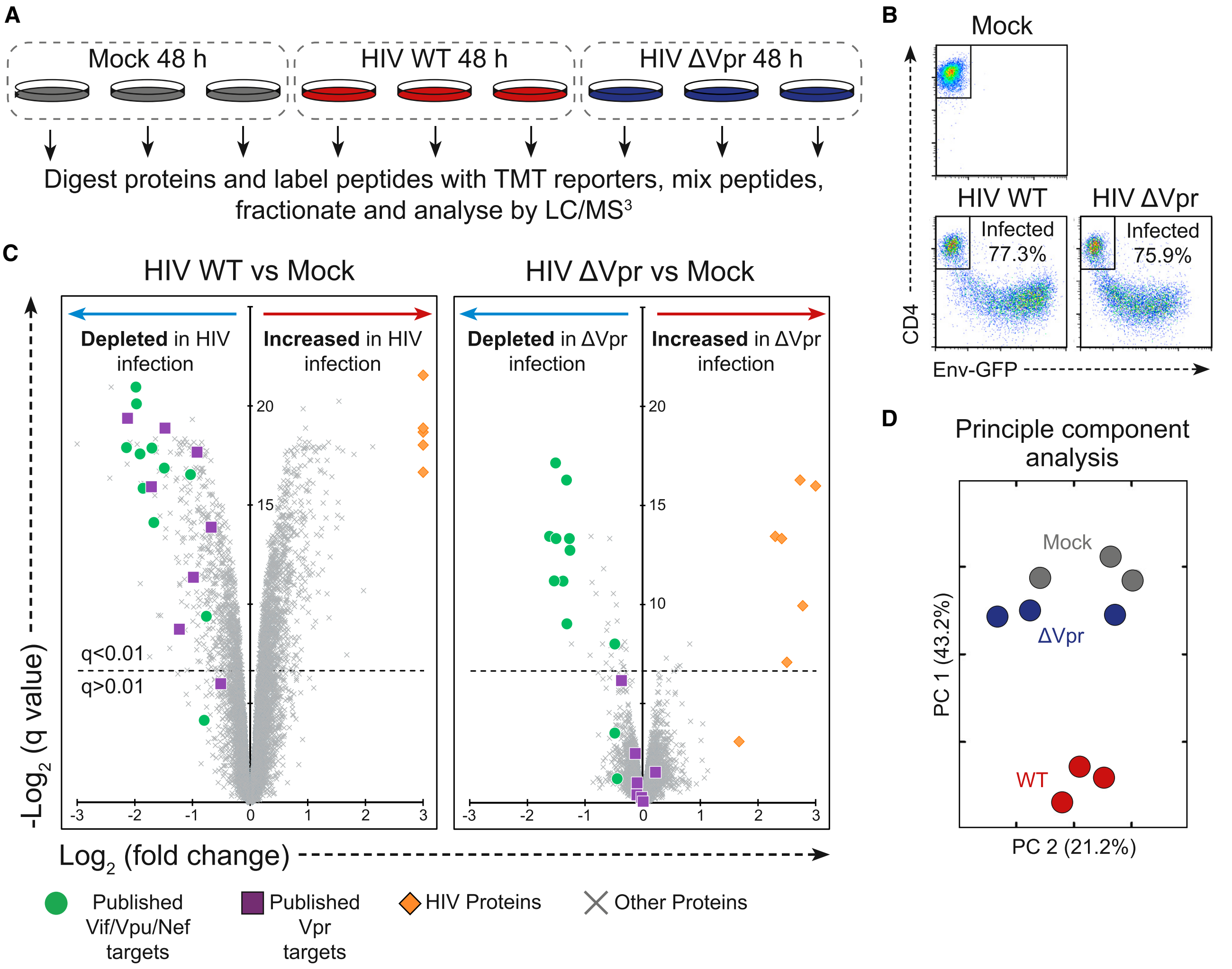

Figure 1. Proteomic Analysis of the Effect of Vpr in HIV Infection

(A) Graphical summary of the HIV and ΔVpr HIV infection TMT experiment.

(B) Fluorescence-activated cell sorting (FACS) plots showing quantification of infection in an example replicate for each of the three conditions. Infected cells lose CD4 expression and become GFP-positive.

(C) Scatterplots displaying pairwise comparisons between wild type, ΔVpr, and mock-infected cells. Each point represents a single protein, with HIV proteins and host proteins of interest highlighted with different symbols (see key).

(D) Principal-component analysis of the samples in this experiment, with wild-type infected (red), ΔVpr (blue), and mock-infected (gray) replicates.

See also Table S1.

the cellular proteome of HIV-1-infected cells and combine this with further unbiased approaches to identify cellular proteins directly targeted and degraded by Vpr. Our data suggest a model for the effects of Vpr on cells in which promiscuous targeting of host factors distinguishes it from other HIV accessory proteins.

RESULTS

Vpr Is Required for Global Proteome Remodeling in HIV-Infected Cells

First, we compared total proteomes of uninfected CEM-T4 T cells with cells infected with either wild-type (WT) HIV or an HIV Vpr deletion mutant (HIV ΔVpr) at an infectious MOI of 1.5 (Figure 1A), resulting in approximately 75% infection (Figure 1B). Data from this experiment are available, together with the other proteomics datasets presented here, in a readily searchable interactive format in Table S1. As expected, among the 7,774 quantitated proteins, we observed widespread changes in cells infected with wild-type HIV (Figure 1C left panel). Together with known Nef, Vpu, and Vif targets, we saw depletion of previously reported Vpr targets, including HLTF (Hrecka et al., 2016; Lahouassa et al., 2016), ZGPAT (Maudet et al., 2013), MCM10 (Romani et al., 2015), UNG (Schröfelbauer et al., 2005), TET2 (Lv et al., 2018), and MUS81 and EME1 (Laguette et al., 2014; Zhou et al., 2016). DCAF1, part of the ligase complex used by Vpr to degrade targets, was also depleted, consistent with a previous report (Lapek et al., 2017).

In HIV ΔVpr infection (Figure 1C, right panel), depletion of Nef, Vpu, and Vif targets was maintained. Remarkably, as well as abolishing depletion of known Vpr targets, almost all of the previously uncharacterized protein changes were also reduced or abolished in HIV ΔVpr infection. Although 1,940 proteins changed significantly ($q < 0.01$) in wild-type HIV-infected cells, only 45 significant changes occurred in cells infected with HIV ΔVpr. Indeed, principal-component analysis showed that cells infected with the HIV ΔVpr virus are more similar on the proteome level to uninfected cell than to cells infected with the wild-type virus (Figure 1D).

Incoming Vpr Protein Alone Drives Global Cellular Proteome Remodeling

Because Vpr enhances the expression of other viral proteins (Forget et al., 1998; Goh et al., 1998; Figure 1C), differences between wild-type and ΔVpr viruses could potentially be explained by secondary changes in expression levels of other proteins or different rates of progression of wild-type and ΔVpr viral infection. To eliminate these potential confounders, we next examined the effect of Vpr acting alone. Unlike other HIV-1 accessory proteins, Vpr is specifically packaged into nascent viral particles. We therefore repeated our proteomics analysis using CEM-T4 T cells exposed to lentiviral particles lacking or bearing Vpr in the presence of reverse transcriptase inhibitors (RTis). This approach excludes all *de novo* viral protein expression, focusing on changes induced by incoming Vpr delivered directly by virions (Figure 2A). For these experiments, cells were exposed to viral particles at an infectious MOI of 0.5 (determined in the absence of RTis).

Strikingly, changes induced by Vpr-containing viral particles phenocopied the Vpr-dependent proteome remodeling seen in HIV infection (Figure 2B) with a high degree of correlation ($r^2 = 0.67$; Figure 2C). Taking both experiments together, Vpr is both necessary and sufficient to cause significant ($q < 0.01$) depletion of at least 302 proteins and upregulation of 413 (highlighted in blue and red, respectively, in Figure 2C). This is a stringent false discovery rate, and, in practice, the number of Vpr-dependent changes is almost certainly even higher. Where antibodies were available, we confirmed a proportion of these changes by immunoblot (Figure 2D).

Cellular Proteome Remodeling Requires Interaction with DCAF1 and Cellular Substrates

Although the function or functions of Vpr remain controversial, in all phenotypic descriptions, Vpr activity is dependent on the interaction between Vpr and the DCAF1/DDB1/Cul4 ligase complex, recruitment of which results in ubiquitination and degradation of known Vpr targets by the ubiquitin-proteasome system (Dehart and Planelles, 2008). Therefore, in addition to testing the effect of wild-type Vpr protein, we tested a number of previously described mutant Vpr variants (Figure 2A). Figure 2E shows an immunoblot of the Vpr protein delivered in the viral particles (top panel) and the abundance of Gag-Pol peptides in the cellular lysate at 48 h determined from the tandem mass tag (TMT) mass spectrometry (MS) experiment. Incoming Vpr alone was sufficient to cause arrest at G2/M, as described previously (Poon et al., 1998), and Figure 2F shows 7-aminoactinomycin D (7-AAD) DNA staining to determine the extent of G2/M arrest under each condition. Visualizations of the proteome remodeling caused by each mutant are shown in Figures 2G and 2H.

Because the Q65 residue of Vpr is required for the interaction with DCAF1 (Le Rouzic et al., 2007), we first compared proteome changes caused by a Q65R Vpr mutant with wild-type Vpr. As predicted, Q65R Vpr was almost completely inactive (Figures 2G and 2H). We recapitulated this finding by comparing the effects of wild-type Vpr in control cells or cells depleted of DCAF1 (Figure S1A). Short hairpin RNA (shRNA)-mediated depletion of DCAF1 resulted in an approximately 50% reduction in protein abundance of DCAF1 (Figure S1B). Because a proportion of cellular DCAF1 was still expressed, known Vpr effects, including degradation of HLTF and upregulation of CCNB1, were partially rather than completely inhibited (Figure S1C). Consistent with this, Vpr-mediated changes were broadly reduced in magnitude in DCAF1 knockdown cells (Figure S1D). Thus, as with depletion of known Vpr targets, extensive Vpr-dependent proteomic remodeling is dependent on the interaction of Vpr with its cognate DCAF1/DDB/Cul4 ligase. Importantly, depletion of DCAF1 alone did not phenocopy Vpr-mediated proteome remodeling, and the widespread effects of Vpr are therefore unlikely to result from sequestration and/or depletion of DCAF1.

Residues E24, R36, Y47, D52, and W54 of Vpr are also required for recruitment and degradation of previously described Vpr targets and have been reported to form the substrate-binding surface (Hrecka et al., 2016; Selig et al., 1997; Wu et al., 2016). In particular, Y47, D52, and W54 make up the proposed DNA-mimicking motif by which Vpr binds the cellular target UNG2 (Wu et al., 2016). In agreement, the $Vpr_{E24R,\ R36P}$ and Vpr_{W54R} mutants showed attenuated remodeling of the proteome, whereas a triple mutant, $Vpr_{Y47A,\ D52A,\ W54R}$, was defective for almost all Vpr-dependent protein changes (Figures 2G and 2H). Global protein remodeling therefore depends on both the substrate binding surfaces of Vpr and recruitment of DCAF1, suggesting that this process is mediated by recruitment of Vpr substrates to the DCAF1/DDB1/CUL4 E3 ligase complex and their subsequent degradation.

Vpr causes G2/M arrest in cycling cells, but the mechanism remains contentious (Belzile et al., 2010; Berger et al., 2015; Fregoso and Emerman, 2016; Goh et al., 1998; Höhne et al., 2016; Laguette et al., 2014; Liang et al., 2015; Re et al., 1995; Romani et al., 2015; Terada and Yasuda, 2006), as is the connection to the replicative advantage Vpr provides *in vivo*. To investigate this important issue, we took advantage of previously characterized Vpr mutants. Residue S79 of Vpr is required for Vpr-dependent cell cycle arrest (Zhou and Ratner, 2000; Figure 2F). Of the other mutants we tested, Vpr_{Q65R} and $Vpr_{Y47A,\ D52A,\ W54R}$ are also unable to cause G2/M arrest, $Vpr_{E24R,\ R36P}$ had an intermediate phenotype, and Vpr_{W54R} caused G2/M arrest at wild-type levels (Figure 2F). Strikingly, most Vpr-dependent protein changes were also observed with the Vpr_{S79A} mutant (Figures 2G and 2H) and are therefore independent of G2/M cell cycle arrest. The presence or absence of G2/M arrest was also a poor correlate of proteomic remodeling across the entire panel of mutants. Cell cycle arrest therefore only explains a minority of Vpr-dependent changes (Figures 2G and 2H).

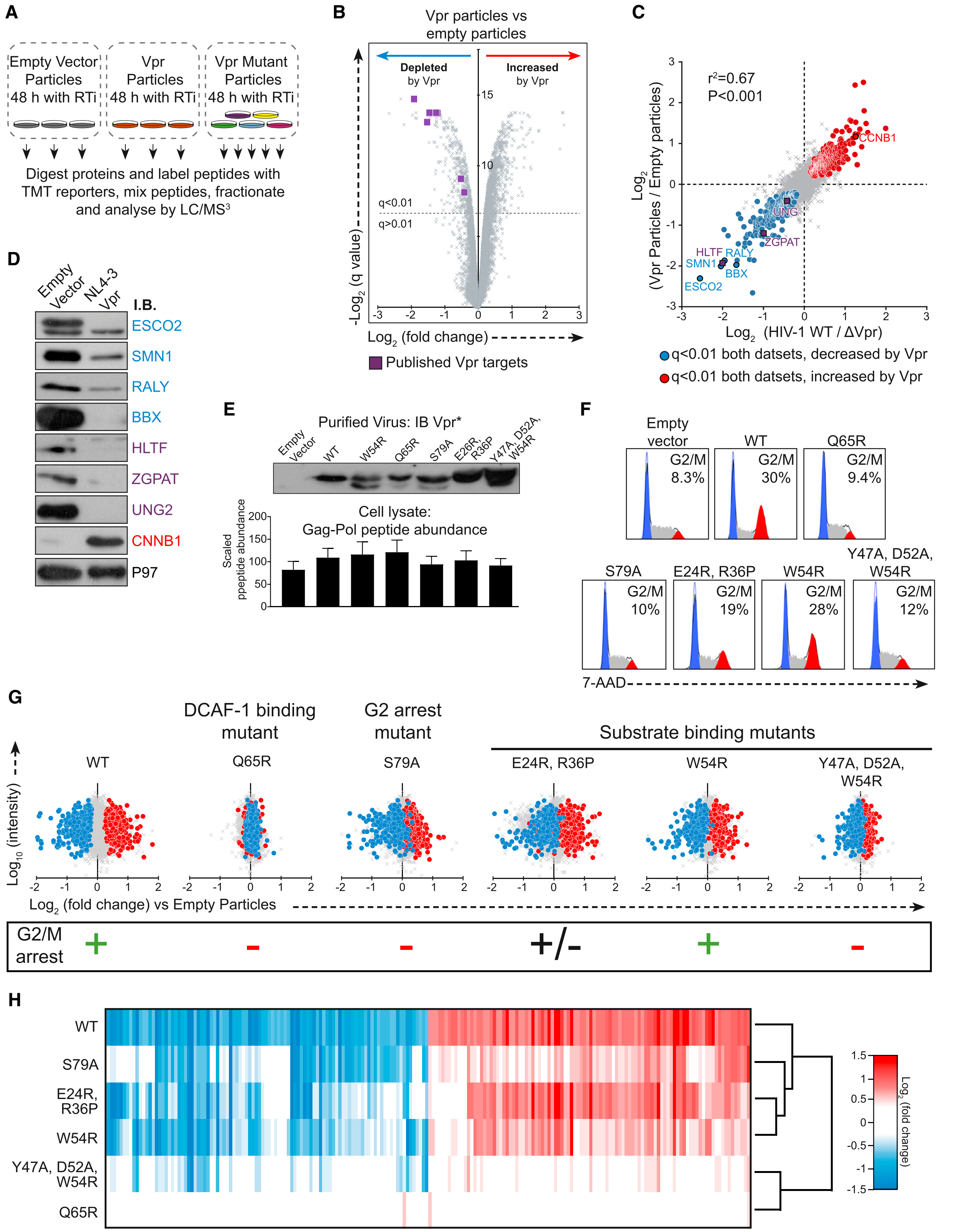

(legend on next page)

To confirm this finding, we examined published datasets describing proteins increased or depleted during different phases of the cell cycle or in chemically G2/M-arrested cells (Fischer et al., 2016; Ly et al., 2015; Figure S2). Compared with cells exposed to Vpr in our study, cells arrested in G2 using a PLK1 inhibitor showed similar regulation of the cyclin family of proteins (Figure S2B), but there was little other correlation between these datasets. Thus, although some changes in protein levels induced by Vpr may be explained by the effects of cell cycle arrest, proteins regulated by the cell cycle in these datasets only account for a minority of Vpr-dependent changes (Figures S2A, S2C, and S2D).

Vpr Directly Targets Multiple Nuclear Proteins with Nucleic Acid Binding Activity

Vpr has nuclear localization, and all reported direct Vpr targets are nuclear proteins. Primary Vpr targets are therefore predicted to be nuclear. Conversely, secondary effects resulting from, for example, transcriptional changes should be distributed across the cell. Analysis of the 302 proteins depleted by Vpr revealed profound enrichment for proteins that reside in the nucleus (>80%) (Figure 3A, left). This raised the possibility that a significant proportion of proteins depleted by Vpr are directly targeted by Vpr because secondary effects should not be limited to the nucleus. Consistent with this hypothesis, proteins upregulated by Vpr (Figure 3A, right), which are all predicted to be secondary indirect effects, were distributed across multiple compartments. Furthermore, proteins depleted by Vpr were enriched (>70%) for nucleic acid binding activity (Figure 3B, left). Vpr associates with DNA-binding proteins such as UNG via a substrate-binding surface that mimics DNA (Wu et al., 2016). Thus, rather than targeting a small number of cellular proteins for degradation, Vpr may have a much wider range of direct targets, and the structure of the substrate binding surface suggests a possible mechanism for promiscuous recruitment of DNA- and RNA-binding cellular proteins.

To identify proteins targeted directly by Vpr, we first adopted a co-immunoprecipitation approach (Figure 3C). CEM-T4 T cells were transduced with a 3× hemagglutinin (HA)-tagged Vpr lentivirus in the presence of an shRNA to DCAF1 and the pan-cullin inhibitor MLN4924 to minimize substrate degradation and enhance co-immunoprecipitation. Factors specifically co-immunoprecipitated in the presence of Vpr are expected to include direct Vpr targets and, accordingly, were enriched for proteins depleted (rather than increased) in the presence of Vpr (Figures 3D–3G). However, the co-immunoprecipitation (coIP) was dominated by DCAF1, a stable binding partner of Vpr, identified with a signal intensity 2 orders of magnitude greater than that of all other proteins (Figure 3E). This is despite knockdown of DCAF1 in these cells, which reduces the DCAF1 protein abundance by approximately 50% (Figure S2B). In addition, at least 13 proteins co-immunoprecipitating with Vpr here have been reported previously to physically interact with DCAF1 alone (Coyaud et al., 2018; Guo et al., 2016; Hossain et al., 2017), of which 11 are not regulated by Vpr, and two, CEP78 and IQGAP2, are upregulated by Vpr, explaining their presence in this list of proteins.

This mismatch between the high abundance of DCAF1 and the relatively low abundance of direct Vpr targets for degradation is consistent with previous reports that have also found that IP-MS based techniques are ideal for identification of the cellular machinery co-opted by viral proteins but often struggle to identify cellular targets that interact transiently and in competition with each other (Jäger et al., 2011; Luo et al., 2016). We therefore adopted an alternative approach, pulsed-stable isotope labeling with amino acids in cell culture (pulsed SILAC), to identify host proteins specifically destabilized within 6 h of exposure to Vpr (Figure 4A). This technique is directly analogous to a traditional pulse-chase experiment using radiolabeled methionine and/or cysteine but allows a global unbiased analysis of potential cellular targets (Boisvert et al., 2012). Because proteins are fully labeled prior to exposure to Vpr, differences in abundance of labeled proteins between conditions exclusively reflect changes in protein degradation rates.

Six hours after exposure to Vpr, the stability of most proteins was unchanged (Figure 4B, top panel). However, a subset of proteins depleted by Vpr was already destabilized, consistent with Vpr-dependent proteasomal degradation. Therefore, these 27 proteins, including HLTF, represent direct targets for Vpr-mediated depletion (Figure 4C). After 24 h of exposure to Vpr,

Figure 2. Analysis of the Nature of Vpr-Mediated Proteome Remodeling

(A) Graphical summary of the Vpr viral particle TMT experiment. Three replicates of cells exposed to empty viral particles or Vpr-bearing viral particles along with single replicates of cells exposed to viral particles bearing five different Vpr mutants were analyzed.

(B) Scatterplot displaying pairwise comparison between cells exposed to empty or Vpr-bearing viral particles.

(C). Scatterplot comparing pairwise comparisons from two proteomics experiments, demonstrating the effect of Vpr in the context of HIV-1 infection (x axis, as shown in Figure 1A) or through cellular exposure to Vpr protein alone (y axis, as shown in Figure 2A). Labeled proteins were selected for confirmation by immunoblot in (D).

(D) Immunoblot of selected proteins in CEM-T4 T cells transduced with an empty vector lentivirus or Vpr-encoding lentivirus 48 h after transduction.

(E) Top: immunoblot of purified virus preparations used to infect cells for the proteomics experiment displayed in (A). Changes to amino acid sequence may reduce the affinity of antibody binding. Bottom: bar chart showing the average scaled abundance of matrix, capsid, and integrase peptides detected in the cell lysate by MS. Bars show mean and SD.

(F) 7-AAD stain of cells exposed to empty vector, Vpr wild-type, or Vpr mutants. Watson pragmatic modeling was used to identify cells in G1 (blue), S (gray), or G2/M (red) phase.

(G) Scatterplots showing pairwise comparison of each Vpr mutant tested and empty vector control, with defined groups of 302 Vpr-depleted and 413 increased proteins highlighted in blue and red, respectively.

(H) Heatmap showing the behavior of the 100 proteins most depleted by Vpr particles (blue) and increased (red) within the defined significantly modulated subsets. Color indicates the $\log_2$ fold change of each protein in each condition compared with empty-particle treatment. Genes were clustered using uncentered Pearson correlation and centroid linkage, and conditions were clustered by column means.

See also Figures S1 and S2.

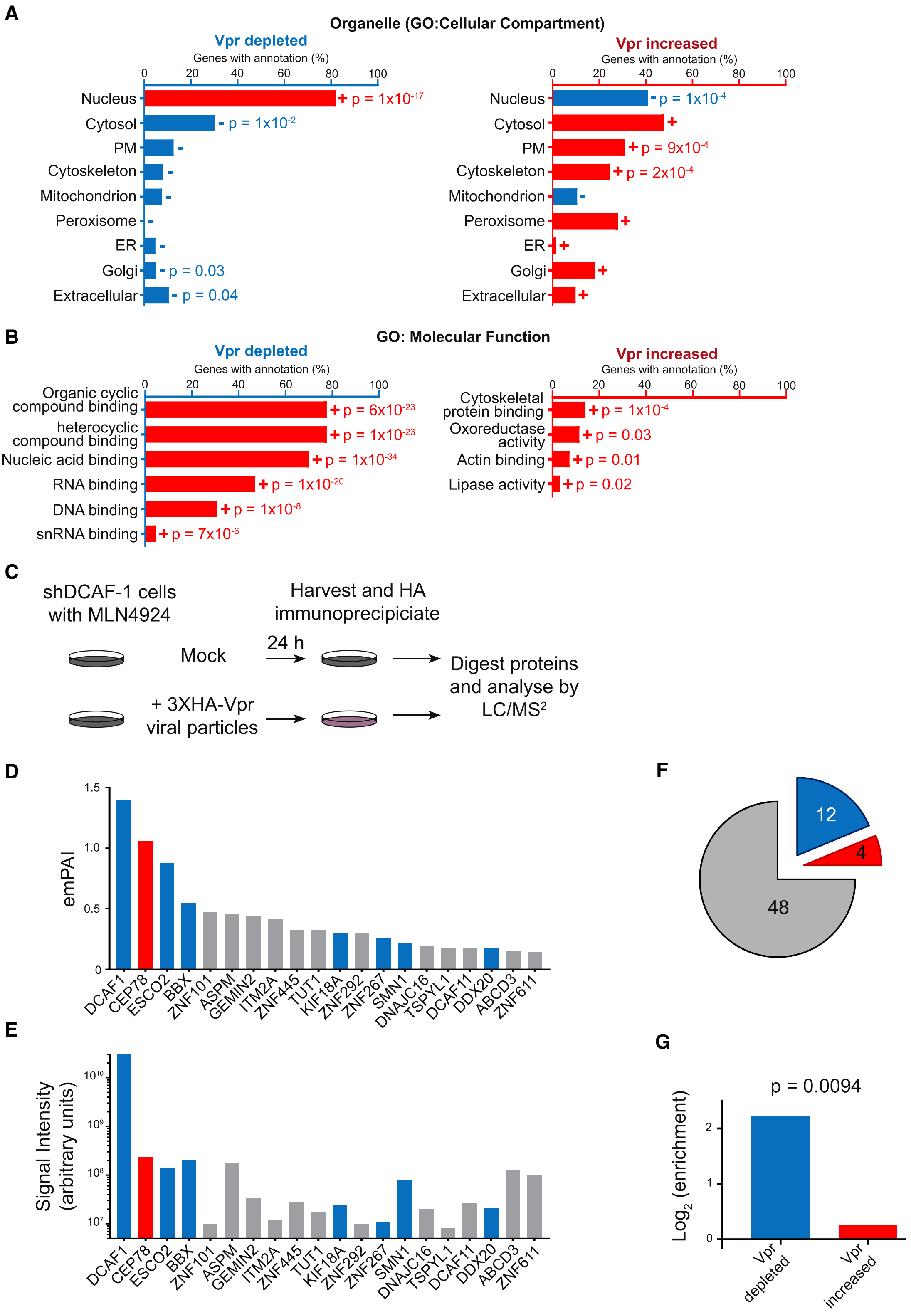

Figure 3. Co-immunoprecipitation MS to Identify Direct Targets for Vpr-Mediated Degradation

(A) Defined groups of Vpr-depleted and increased proteins were subject to gene ontology enrichment analysis and compared with a background of all proteins quantitated in these experiments. Gene Ontology (GO) cellular compartment enrichment analysis results were manually curated for 9 commonly used organelle

(legend continued on next page)

changes in protein stability reflected overall changes in protein abundance caused by Vpr in other experiments (Figure 4B, bottom panel), including proteins with increased as well as decreased stability. These changes are therefore indicative of both direct and indirect Vpr targets.

Combining all orthogonal approaches—whole cell proteomics to identify proteins depleted by Vpr in the context of viral infection or Vpr protein alone delivered in viral particles, MS coIP with epitope-tagged Vpr, and pulsed SILAC-based identification of proteins post-translationally degraded by Vpr—we identified at least 38 direct targets for Vpr-dependent degradation (Table 1). Vpr is both necessary and sufficient for depletion of these proteins, which are either bound by Vpr, or destabilized within 6 h of Vpr exposure (or both). In practice, this list very likely underestimates the true number of direct Vpr targets because several known targets of Vpr behaved appropriately but beyond the statistical cutoffs used to derive this table (Table S2). Table 1 is also limited to proteins expressed in the CEM-T4 T cell model, which excludes some known Vpr targets such as SMUG1 (Schröfelbauer et al., 2005). It should be noted that some cellular phenotypes for Vpr are exclusive to non-T cell targets, such as primary dendritic cells (Miller et al., 2017) and macrophages (Connor et al., 1995; Mashiba et al., 2015), and are unlikely to be explained by Vpr-mediated depletion of any of the proteins listed here.

The Direct Vpr Targets SMN1, CDCA2, and ZNF267 Contribute to G2/M Cell Cycle Arrest

Several cellular phenotypes have been described for Vpr, including G2/M arrest, transactivation of the HIV long terminal repeat (LTR), and modulation of cellular signaling pathways such as NFκB and NFAT arrest (Bolton and Lenardo, 2007; Gummuluru and Emerman, 1999; Höhne et al., 2016; Liang et al., 2015; Liu et al., 2014; Muthumani et al., 2006; Rogel et al., 1995). The mechanisms responsible for these phenotypes are controversial. Wide-scale proteome remodeling by Vpr and direct targeting of multiple proteins suggest a model in which Vpr interacts with diverse cellular proteins and pathways, resulting in cumulative or redundant effects on cellular phenotypes. This model does not contradict any single mechanism but suggests that several are involved, with potential variability between different cell types and experimental systems.

To test our model, we investigated the best-described phenotype for Vpr, cell cycle arrest at the G2/M phase. We hypothesized that differential depletion of cellular proteins by different Vpr mutants tested in Figure 2, which displayed a spectrum of capacity to cause G2/M arrest, would highlight proteins whose depletion results in this cellular phenotype. We first examined proteins with a published connection to Vpr-mediated G2/M arrest: MCM10, MUS81, and EME1. MCM10 has been reported to be directly degraded by Vpr, resulting in cell cycle arrest (Romani et al., 2015). Vpr-mediated depletion of MUS81 and EME1 has been proposed to be a consequence of Vpr interaction with SLX4 (Laguette et al., 2014), another proposed mechanism of Vpr-mediated G2/M arrest, although this is controversial (Berger et al., 2015; Fregoso and Emerman, 2016; Zhou et al., 2016). In our system, of these three proteins (MCM10, MUS81, and EME1), only depletion of MCM10 showed a strong correlation with the extent of G2/M arrest caused by the different mutants (Figure 5A). As described previously (Romani et al., 2015), depletion of MCM10 by RNAi was sufficient to cause accumulation of CEM-T4 T cells at G2/M (Figures 5B and 5C).

We therefore interrogated our Vpr mutant dataset (Figure 2) for other direct Vpr targets (Table 1) that, like MCM10, correlated with the extent of G2/M arrest. In total, we identified 14 targets with a significant relationship ($p < 0.05$ in a linear regression analysis) (Figure 5D). Next we tested whether shRNA-mediated depletion could phenocopy Vpr-dependent cell cycle arrest at G2/M (Figure 5D). Depletion of 3 Vpr targets (SMN1, CDCA2, and ZNF267) caused G2/M arrest (Figures 5D and 5E). The correlation between G2/M arrest and depletion of these three proteins by Vpr mutants is shown in Figure 5F. The phenotype resulting from RNAi protein depletion was confirmed with a second shRNA (Figure 5G). Thus, several Vpr targets contribute independently to G2/M cell cycle arrest, consistent with a model whereby depletion of multiple cellular proteins underpins the various phenotypes associated with Vpr expression.

Some Vpr Targets Are Conserved across Primate Lentiviruses, but Global Cellular Proteome Remodeling Is Unique to the HIV-1/SIVcpz Lineage

Targeting of key cellular proteins such as BST2 or the APOBEC3 family is conserved across multiple lentiviral lineages, demonstrating the *in vivo* selective advantage of these interactions. We therefore tested a diverse panel of lentiviral Vpr proteins to determine whether they share activity with the NL4-3 Vpr variant used in all of the experiments above. We included Vpr variants from primary isolates of HIV-1 from two distinct cross-species

level classifications, shown here. Bars shown in red with a + symbol were enriched compared with the expected number through chance, whereas bars in blue (−) were de-enriched. Where $p < 0.05$, the associated p value represents the results of a Fisher's exact test with Bonferroni correction.

(B) GO: molecular function analysis of the Vpr-depleted and -increased proteins, in this case showing all terms enriched within each group with $p < 0.05$ in a Fisher's exact test with Bonferroni correction.

(C) Graphical summary of the IP-MS experiment. All cells were stably transduced with a ShDCAF1 vector as described earlier. MLN4924 is a pan-Cullin inhibitor.

(D) 20 most abundant proteins identified by coIP determined by number of unique peptides, normalized as a proportion of the maximum possible peptide count for each protein (exponentially modified protein abundance index [emPAI]). Proteins falling within the defined list of 302 Vpr-depleted and 413 Vpr-increased proteins are highlighted in blue and red, respectively.

(E) The same 20 proteins with signal intensity rather than peptide count shown.

(F) Pie chart indicating the overlap between the proteins co-immunoprecipitated with Vpr and the defined list of 302 Vpr-decreased (blue) and 413 Vpr-increased proteins (red) and proteins detected but falling into neither list (gray).

(G) Bar chart showing the enrichment of Vpr-depleted and Vpr-increased proteins within proteins co-immunoprecipitated with Vpr compared with the expected numbers of proteins that would be co-immunoprecipitated from each group by chance. The p value was calculated by Fisher's exact test of a 2 × 2 contingency table (Vpr-depleted or -increased, identified by coIP or not identified).

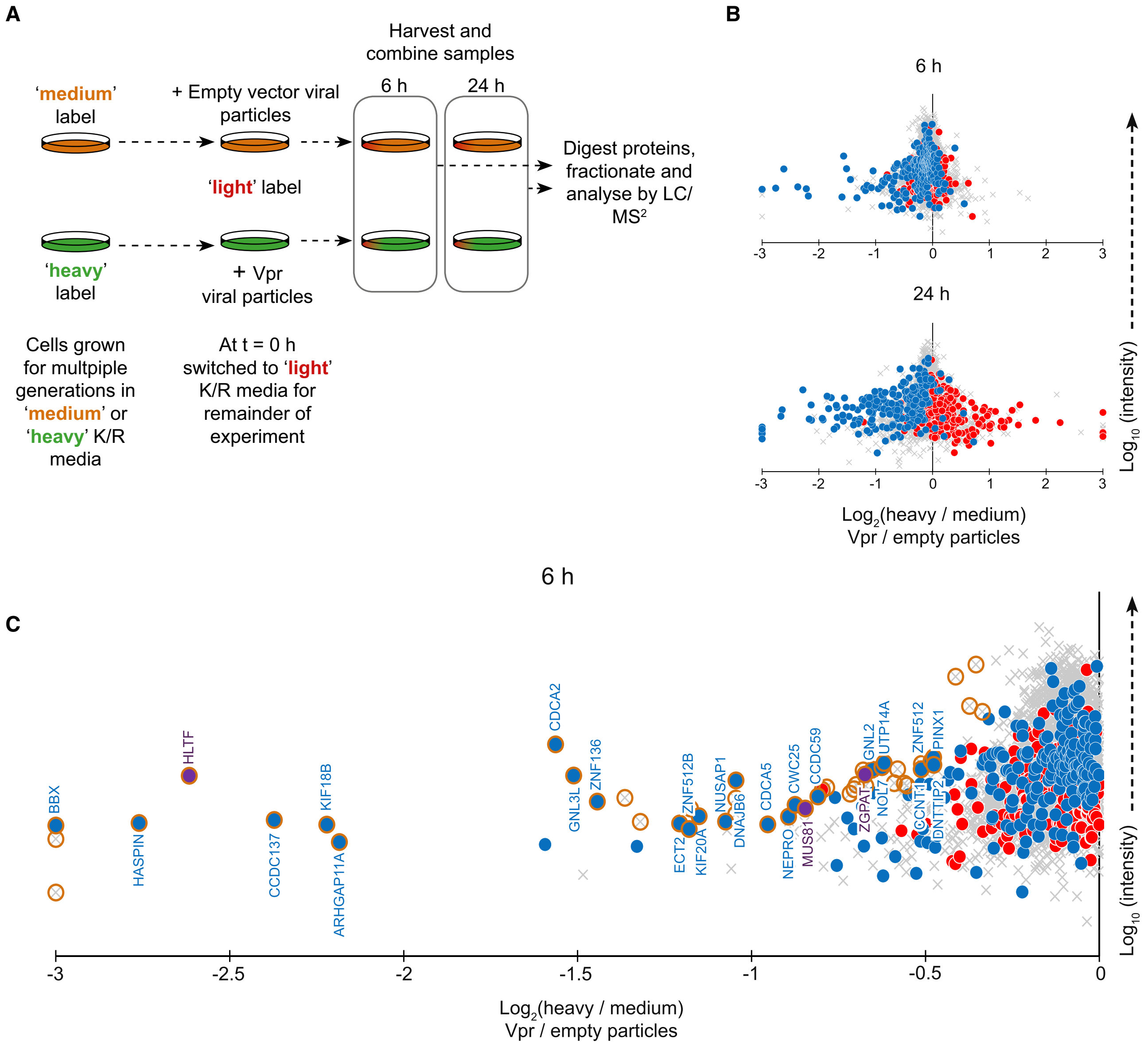

Figure 4. Pulsed SILAC Method to Identify Direct Targets for Vpr-Mediated Degradation

(A) Graphical summary of the pulsed SILAC experiment.

(B) Scatterplots showing the changes to protein stability of proteins after 6 or 24 h of exposure to a Vpr-bearing lentivirus compared with a control lentivirus, with previously defined groups of Vpr-depleted (blue) and -increased (red) proteins highlighted.

(C) Expanded view of proteins degraded within 6 h of Vpr exposure. Significantly degraded (significance B [Sig.B] < 0.01) proteins are highlighted in gold. The previously described Vpr targets HLTF, MUS81, and ZGPAT are shown in purple.

transmissions from apes to humans (group M and group O), in addition to a closely related variant from Simian immunodeficiency virus (SIV) of chimpanzees (SIVcpz). We also tested Vpr variants from divergent primate lineages, including HIV-2, and SIVs from sooty mangabeys (SIVsmm), African green monkeys (SIVagm), and red-capped mangabeys (SIVrcm) (Figures 6A–6C). In addition to Vpr, which is present in all primate lentiviruses, viruses of some lineages also bear Vpx, a gene duplication of Vpr. Because depletion of some substrates and cellular functions switches between Vpr and Vpx in lineages encoding this accessory gene (Fletcher et al., 1996; Lim et al., 2012), we also included a Vpx variant from HIV-2.

Extensive Vpr-dependent remodeling of the cellular proteome was conserved across the HIV-1/SIVcpz lineage (Figure 6D, top row). Although Vpr variants from other lineages showed a narrower set of changes, depletion of some proteins, particularly those most heavily depleted by HIV-1 Vpr, was conserved across multiple lineages (Table 2). Depletion of selected proteins for which commercial antibody reagents were available was readily confirmed by immunoblot of cells transduced with an

Table 1. Direct Targets for Vpr-Mediated Degradation

Accession	Gene	Previously Confirmed Direct Target	Predicted from Temporal Profile[a]	Vpr Necessary (Figure 1)	Incoming Vpr Sufficient (Figure 2)	Degraded within 6 h (Figure 4)	CoIP (Figure 3)
Q56NI9	ESCO2	–	–	yes	yes	ND	yes
Q16637	SMN1 / SMN2	–	yes	yes	yes	NS	yes
Q14527	HLTF	yes[b]	yes	yes	yes	yes	–
Q96KM6	ZNF512B	–	–	yes	yes	yes	–
Q8WY36	BBX	–	–	yes	yes	yes	yes
A6NFI3	ZNF316	–	–	yes	yes	yes	–
Q8NI77	KIF18A	–	–	yes	yes	ND	yes
Q8TF76	HASPIN	–	–	yes	yes	yes	–
Q9Y4B6	VPRBP	yes[c]	–	yes	yes	NS	yes
Q96FF9	CDCA5	–	yes	yes	yes	yes	–
Q6PK04	CCDC137	–	yes	yes	yes	yes	–
Q9NXE8	CWC25	–	–	yes	yes	yes	–
Q86Y91	KIF18B	–	yes	yes	yes	yes	–
Q6P4F7	ARHGAP11A	–	–	yes	yes	yes	–
Q6NW34	NEPRO	–	–	yes	yes	yes	–
Q9BVJ6	UTP14A	–	–	yes	yes	yes	–
Q9NVN8	GNL3L	–	yes	yes	yes	yes	–
Q5QJE6	DNTTIP2	–	–	yes	yes	yes	–
Q69YH5	CDCA2	–	–	yes	yes	yes	–
Q96BK5	PINX1	–	–	yes	yes	yes	–
Q8N5A5	ZGPAT	yes[d]	–	yes	yes	yes	–
Q13823	GNL2	–	–	yes	yes	yes	–
Q9UHI6	DDX20	–	–	yes	yes	NS	yes
Q96ME7	ZNF512	–	–	yes	yes	yes	–
Q14586	ZNF267	–	–	yes	yes	NS	yes
Q9H8V3	ECT2	–	–	yes	yes	yes	–
P57678	GEMIN4	–	–	yes	yes	NS	yes
P52701	MSH6	–	–	yes	yes	NS	yes
O75190	DNAJB6	–	–	yes	yes	yes	–
Q96NY9	MUS81	yes[e]	–	yes	yes	yes	–
O60563	CCNT1	–	–	yes	yes	yes	–
Q9P031	CCDC59	–	–	yes	yes	yes	–
Q9UIS9	MBD1	–	–	yes	yes	NS	yes
Q9UMY1	NOL7	–	–	yes	yes	yes	–
Q6ZN55	ZNF574	–	–	yes	yes	NS	yes
Q9BXS6	NUSAP1	–	–	yes	yes	yes	–
O95235	KIF20A	–	–	yes	yes	yes	–
P35251	RFC1	–	–	yes	yes	NS	yes

ND, not detected or quantitated in this experiment; NS, degraded but with Sig.B > 0.01. See also Table S2.
[a]Proteins previously predicted as potential Vpr targets because of a similar pattern of temporal regulation in HIV-1 infection (Greenwood et al., 2016).
[b]Hrecka et al., 2016; Lahouassa et al., 2016.
[c]Lapek et al., 2017.
[d]Maudet et al., 2013.
[e]Laguette et al., 2014; Zhou et al., 2016.

overlapping panel of Vpr variants (Figure 6E). These conserved targets of direct Vpr-mediated degradation are likely to provide an *in vivo* replicative advantage for all primate lentiviruses. Although the failure of SIVrcm, SIVagm, or SIVsmm Vpr variants to degrade proteins that are targeted by HIV-1/SIVcpz may be due to the use of human rather than cognate primate cells, it is

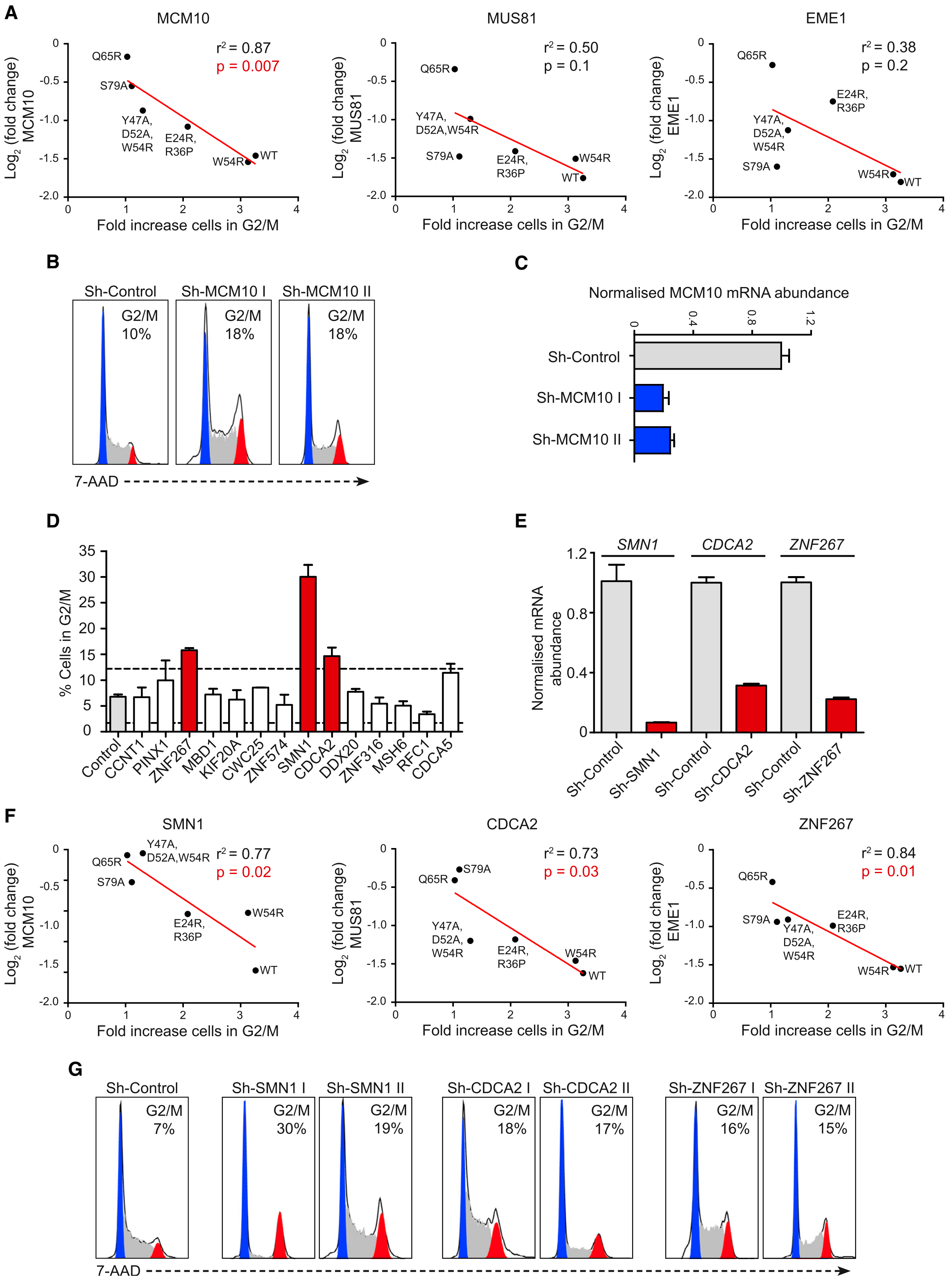

(legend on next page)

notable that HIV-2 Vpr did not cause global proteome remodeling, despite being tested in the host cell to which it is adapted.

Although none of the identified HIV-1 Vpr targets were degraded by the HIV-2 Vpx (HIV-2_{ROD}) tested, we noted a shared ability of HIV-2 Vpx and SIVagm Vpr to deplete TASOR, a critical component of the human silencing hub (HuSH) transcription repressor complex (Tchasovnikarova et al., 2015; Figure 7A). While this manuscript was in preparation, two other groups independently discovered and reported Vpx-mediated depletion of TASOR (Chougui et al., 2018; Yurkovetskiy et al., 2018). The HuSH complex mediates position-dependent transcriptional repression of a subset of lentiviral integrations, and we showed previously that antagonism of HuSH is able to potentiate HIV reactivation in the J-LAT model of latency (Tchasovnikarova et al., 2015). As predicted, Vpx phenocopied the effect of RNAi-mediated TASOR depletion on reactivation of the HuSH-sensitive J-LAT clone A1 (Figure 7B).

Previous reports (Chougui et al., 2018; Yurkovetskiy et al., 2018) were conflicted regarding the conservation of TASOR depletion across Vpr variants. With some exceptions, most primate lentiviruses can be categorized into 5 lineages (Figure 7C), two of which encode Vpx. The previously described canonical function of Vpx is degradation of SAMHD1 (Hrecka et al., 2011; Laguette et al., 2011). Two lentiviral lineages lack Vpx but use Vpr to degrade SAMHD1, whereas the HIV-1/SIVcpz lineage lacks SAMHD1 antagonism (Lim et al., 2012). We considered that TASOR antagonism may follow the same pattern and thus tested Vpx proteins from both Vpx-bearing lineages and representative Vpr variants from lineages that use Vpr to degrade SAMHD1. All of these proteins were able to deplete TASOR in Vpx/Vpr-transduced cells (Figure 7D), and, therefore, antagonism of SAMHD1 and TASOR appears to follow the same pattern.

Although the targeting of some substrates was conserved across Vpr variants from multiple lineages, we did not observe broad proteome remodeling outside of the HIV-1/SIVcpz lineage. However, in the experiment described in Figure 6A, an HIV-1-based lentiviral transduction system was used. Of the Vpr and Vpx variants tested, only Vpr proteins from the HIV-1/SIVcpz alleles are efficiently packaged into the virion. In these cases, cells receive both incoming and *de novo*-synthesized Vpr, whereas, in the case of other variants tested, only *de novo*-synthesized Vpr is present. Therefore, there is a time lag of 18–24 h for viral entry, reverse transcription, integration, and *de novo* synthesis of protein to begin. The more limited proteome remodeling seen in Vpr variants could reflect a lack of time for such changes to occur.

To account for this, we carried out an experiment in which cells were transduced with Vpr or Vpx from the primary HIV-2 isolate 7312a and assayed 48–96 h post-transduction (Figure 7E), allowing time for additional changes to develop after *de novo* Vpr/Vpx synthesis. Even 96 h after transduction, HIV-2 Vpr showed very limited changes. The majority of these changes consisted of depletion of proteins also targeted by HIV-1 Vpr (Figure 7F, left panel). Curiously, 7312a HIV-2 Vpx also depleted several proteins modulated by HIV-1 Vpr (Figure 7F, right panel), including direct targets for HIV-1 Vpr-mediated degradation, BBX, HASPIN, and ARHGAP11A. Some proteins were degraded by both HIV-2 Vpr and Vpx, whereas others were only degraded by the Vpx of this isolate. In lentiviral strains and/or lineages that encode both Vpr and Vpx, the responsibility for degrading certain targets of HIV-1 Vpr is therefore shared between Vpr and Vpx, further emphasizing their *in vivo* importance.

Conversely, global proteome remodeling does not occur from extended expression of HIV-2 Vpr or Vpx. The difference between HIV-1 and HIV-2 Vpr in this respect may contribute, in part, to the increased pathogenicity of HIV-1 in humans over HIV-2 (Jaffar et al., 2004), particularly given the potential for HIV-1 Vpr to drive these changes in Vpr-exposed but uninfected bystander cells.

DISCUSSION

Proteomics analyses of cells infected with viruses from different orders have revealed widespread and varied changes to the cellular proteome (Diamond et al., 2010; Ersing et al., 2017; Greenwood et al., 2016; Weekes et al., 2014). Although these changes are presumed to be multifactorial, in the case of HIV-1 infection, our data show that the majority of changes can be attributed to the action of a single viral protein, Vpr. We propose that this global cellular proteome remodeling consists of direct targeting of multiple cellular proteins for proteasomal degradation via the DCAF1/DDB1/CUL4A E3 ligase complex, followed by resulting secondary effects on other proteins.

Figure 5. Direct Vpr Targets Involved in G2/M Arrest

(A) Correlation between depletion of MCM10, MUS81, and EME1 by each Vpr mutant tested in the experiment shown in Figure 2 and the extent of G2/M arrest caused by that mutant. The red line shows linear regression analysis.

(B) Example DNA staining showing G2/M arrest caused by shRNA-mediated depletion of MCM10, representative of three independent experiments. Watson pragmatic modeling was used to identify cells in G1 (blue), S (gray), or G2/M (red) phase.

(C) Real-time qRT-PCR analysis of MCM10 mRNA abundance in cells transduced with control or MCM10-targeting shRNA. Values were generated using the ΔΔCT method relative to GAPDH mRNA abundance and normalized to the control condition. Bars show mean and SEM of three technical replicates.

(D) Targeted shRNA screen of direct Vpr target proteins identified here whose depletion correlated with G2/M arrest in the experiment detailed in Figure 2. Bars show means and SEM of at least two replicates from more than three independent experiments. Dashed lines show control average ± 3 SDs. The control condition contains combined data from three different control shRNAs.

(E) Real-time qRT-PCR analysis of mRNA abundance in cells transduced with control or targeting shRNA. Values were generated using the ΔΔCT method relative to GAPDH mRNA abundance and normalized to the control condition. Bars show mean and SEM of three technical replicates.

(F) Correlation between depletion of SMN1, CDCA2, and ZNF267 by each Vpr mutant tested in the experiment shown in Figure 2 and the extent of G2/M arrest caused by that mutant.

(G) Example DNA staining showing G2/M arrest caused by shRNA-mediated depletion of SMN1, CDCA2, and ZNF267 using a second independent shRNA; representative of at least two independent experiments.

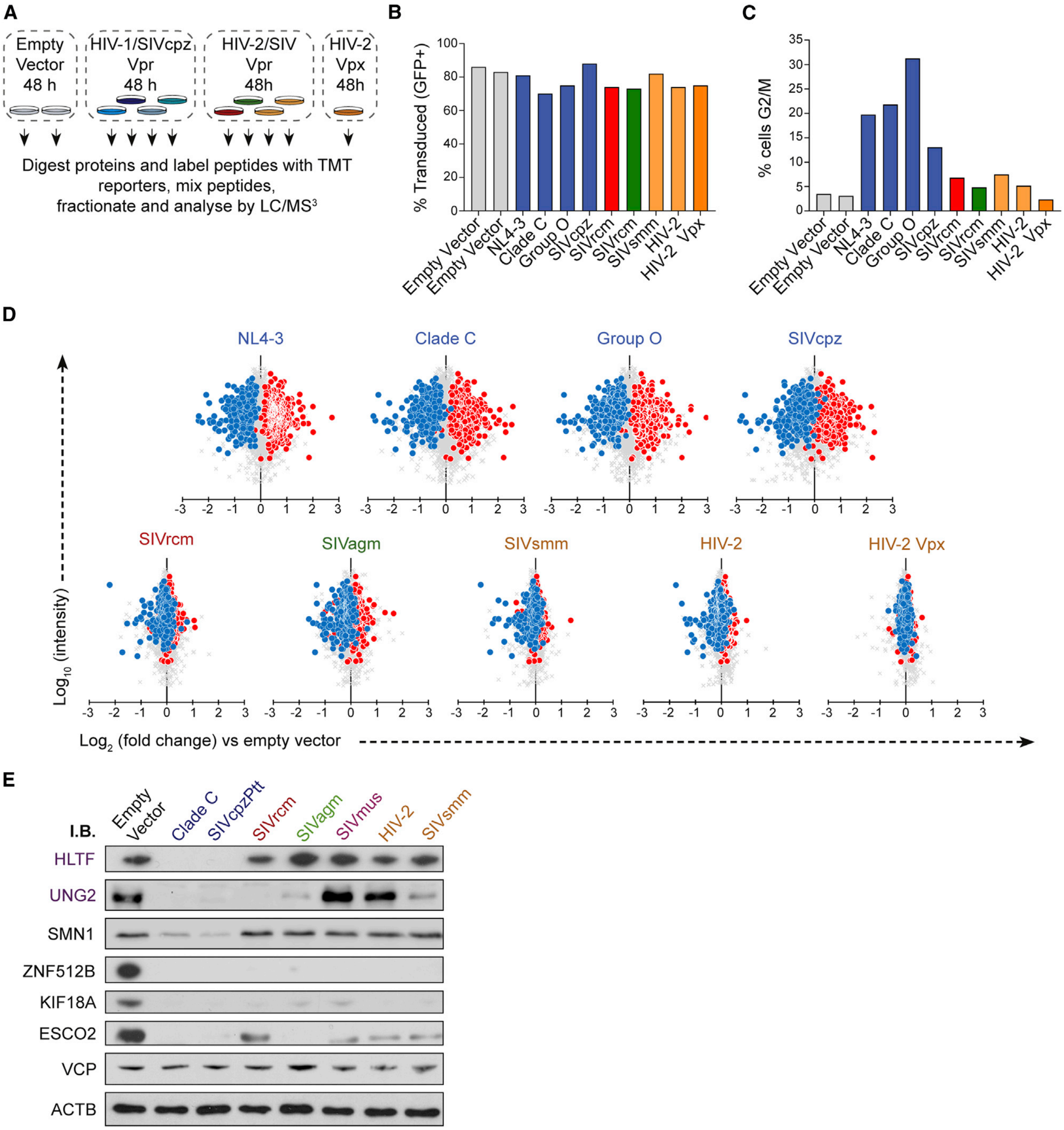

Figure 6. Identification of Proteome Changes Conserved Between Human and Primate Lentiviral Vpr Lineages

(A) Graphical summary of the TMT experiment testing conservation of Vpr function.

(B) GFP+ (transduced) cells at harvest. Cells were transduced at an infectious MOI of 1.5 based on prior titration, with the actual resulting percent transduction varying slightly across the samples. Because non-HIV-1/SIVcpz Vpr is not packaged in the viral particles used, the percent transduced represents all cells exposed to Vpr under those conditions.

(C) Proportion of cells in G2/M at point of harvest, based on 7-AAD staining and Watson pragmatic modeling.

(D) Scatterplots showing the pairwise comparison between each Vpr tested and empty vector control, with defined groups of 302 Vpr-depleted (blue) and 413 increased (red) proteins highlighted.

(E) Immunoblot of example known, non-conserved, and conserved targets of Vpr-mediated depletion. The HIV-2 Vpr is a primary isolate HIV-2 Vpr (7312a), whereas the proteomics experiment described in (A) used HIV-2 ROD Vpr.

Table 2. Proteins Depleted by Vpr Variants from Multiple Lineages

Accession	Gene Name	Direct Target	Lineages Showing Profound Depletion[a]	Profound Depletion by Number of Tested Vpr Variants within Each Lineage			
				HIV-1/SIVcpz	SIVrcm	SIVsmm/HIV-2	SIVagm
Q86Y91	KIF18B	yes	4	4/4	1/1	1/2	1/1
Q8NI77	KIF18A	yes	4	4/4	1/1	2/2	1/1
Q14865	ARID5B	–	4	4/4	1/1	2/2	1/1
P46013	MKI67	–	4	2/4	1/1	2/2	1/1
Q56NI9	ESCO2	yes	3	4/4	0/1	2/2	1/1
Q6P4F7	ARHGAP11A	yes	3	4/4	0/1	1/2	1/1
Q96KM6	ZNF512B	yes	3	4/4	0/1	2/2	1/1
Q9Y4B6	DCAF1	yes	3	4/4	0/1	2/2	1/1
Q6NW34	NEPRO	yes	3	4/4	1/1	2/2	0/1
Q8N3Z6	ZCCHC7	–	3	3/4	0/1	1/2	1/1
Q8NDF8	PAPD5	–	3	2/4	0/1	1/2	1/1
Q8TF76	HASPIN	yes	2	4/4	0/1	0/2	1/1
Q9NXE8	CWC25	yes	2	4/4	0/1	0/2	1/1
Q96BK5	PINX1	yes	2	4/4	0/1	1/2	0/1
Q9NVN8	GNL3L	yes	2	3/4	1/1	0/2	0/1
Q6ZN06	ZNF813	–	2	4/4	1/1	0/2	0/1
Q9NW13	RBM28	–	2	3/4	0/1	1/2	0/1
Q6W2J9	BCOR	–	2	4/4	0/1	0/2	1/1

[a]Defined here as a $\log_2$ fold change of less than -1 compared with the empty vector (50% reduction).

Although many of the changes caused by Vpr are secondary, they occur within the physiological time frame of productive infection (Murray et al., 2011; Perelson et al., 1996) and are therefore relevant to our understanding of the HIV-1-infected cell. Further, we have recently mapped changes to the cellular proteome in HIV-1-infected primary human CD4+ T cells and confirmed that the Vpr-mediated changes described here in CEM-T4s are recapitulated in the natural cell type of HIV infection (Naamati et al., 2019).

Before this study, the list of direct Vpr targets was already extensive. Here we confirmed Vpr-mediated depletion of the previously described Vpr targets HLTF (Hrecka et al., 2016; Lahouassa et al., 2016), ZGPAT (Maudet et al., 2013), MCM10 (Romani et al., 2015), UNG2 (Schröfelbauer et al., 2005), TET2 (Lv et al., 2018), and MUS81 and EME1 (Laguette et al., 2014; Zhou et al., 2016), whereas SMUG1 (Schröfelbauer et al., 2005) and PHF13 (Hofmann et al., 2017) were not detected in our model T cell line. By analogy with other HIV accessory proteins, it might have been predicted that this list of Vpr targets would be nearly complete. Instead, we show here that it is only the tip of the iceberg.

Although surprising, the ability of Vpr to degrade multiple cellular factors may be explained by the biology of this small protein. Mechanistically, depletion of multiple proteins with nucleic acid binding properties is consistent with known structural determinants of Vpr substrate recruitment. From a functional viewpoint, although HIV-1 has three accessory proteins (Vpu, Nef, and Vif) to aid viral replication and counteract host defenses in the late stages of the viral replication cycle, Vpr is the only HIV-1 accessory protein packaged into virions. Multiple targets may therefore be required to protect incoming virions from cellular factors and to prime newly infected cells for productive viral replication.

This work is not the first attempt to use unbiased proteomics analysis to characterize Vpr function. Previous studies have used single proteomics experiments or methods to identify candidate proteins that either interact with or are depleted by Vpr, with individual proteins followed up using targeted immunoreagents. For example, Vpr binding partners were identified by Jäger et al. (2011) and Hrecka et al. (2016) using IP-MS. Jäger et al. (2011) did not determine whether any of these proteins were depleted, whereas Hrecka et al. (2016) focused on the single target protein, HLTF and found it to be depleted by Vpr. Similarly, Lahouassa et al. (2016) used a SILAC-based approach to quantify proteomic changes in cells exposed to viral particles bearing Vpr, identifying 8 proteins that were depleted by at least 20%. Of these, only one, HLTF, was confirmed to be a direct Vpr target by targeted orthogonal approaches.

As expected, the lists of "candidate" Vpr proteins identified but not pursued in the above studies overlaps with this work, and, in some instances, we confirmed these candidates to be direct targets for Vpr-mediated degradation. For example, SMN1 was found to bind Vpr by Jäger et al. (2011) and to be degraded by Vpr by Lahouassa et al. (2016) in independent proteomics experiments. Similarly, ESCO2 was found to bind Vpr by Hrecka et al. (2016), but the depletion was not confirmed by immunoblot, most likely because of poor performance of the commercial antibody used. We have shown here that both of these proteins are direct targets for Vpr-mediated degradation.

OPEN ACCESS
CellPress

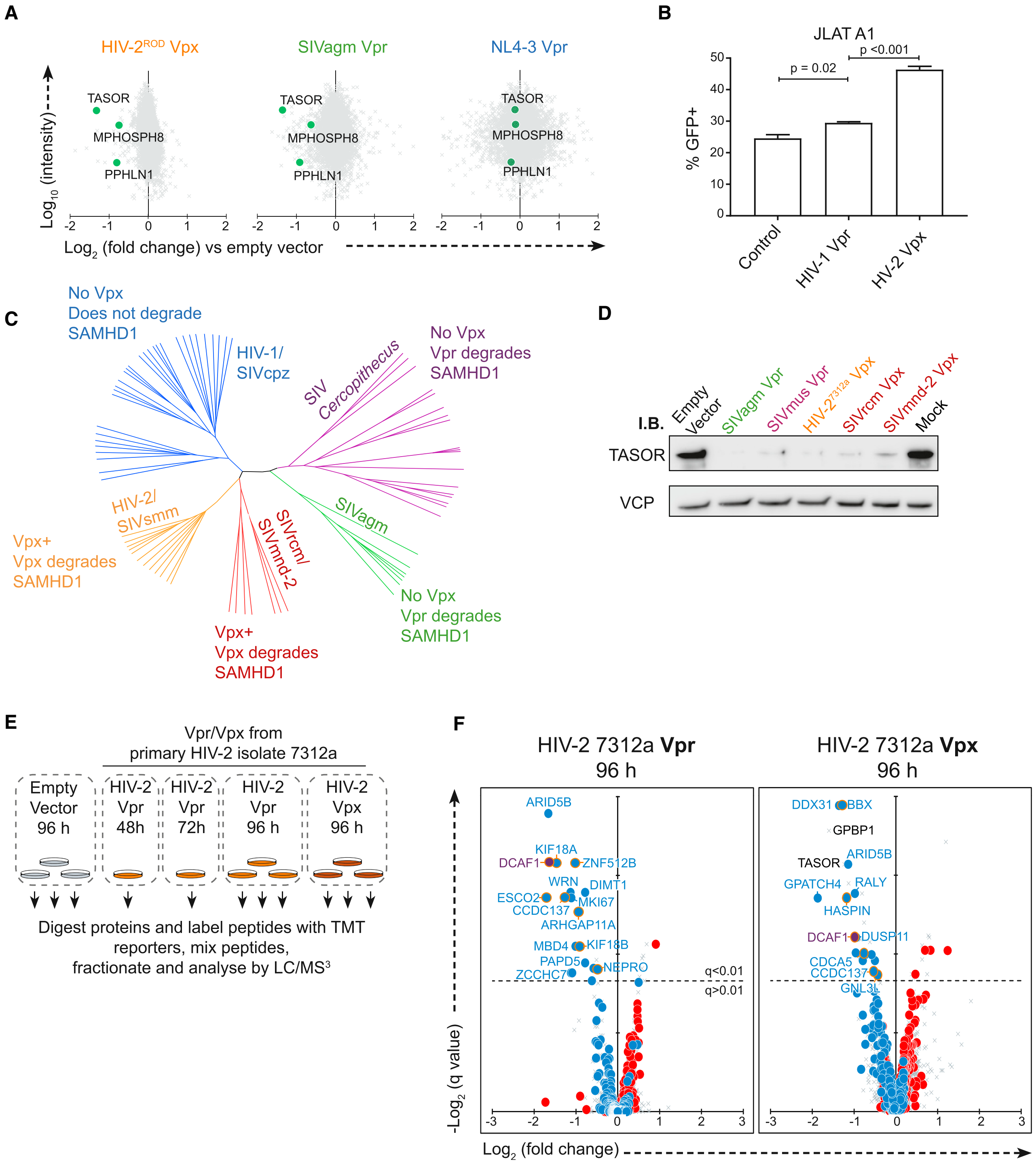

Figure 7. Shared Targets of Lentiviral Vpr and Vpx Proteins

(A) Scatterplots showing pairwise comparison between each Vpr tested and empty vector control, with HuSH complex components highlighted.

(B) Bar graph of the percentage of GFP-positive JLAT-A1 cells after transduction with control (Cre recombinase), Vpr, or Vpx proteins and treatment with tumor necrosis factor alpha (TNF-α). Mean and SEM of 3 biological replicates per condition are shown, representative of three independent similar experiments. The p values were determined by ordinary one-way ANOVA with Bonferroni comparison between Vpr/Vpx treatment and control-treated cells.

(C) Phylogenetic tree of primate lentiviruses based on an alignment of Vpr nucleic acid sequences, with 5 major lineages of primate lentiviruses labeled. Information regarding Vpr and Vpx activity is based on a selected number of isolates tested in each lineage (Lim et al., 2012).

(D) Immunoblot of TASOR in cells transduced with a panel of Vpx and Vpr proteins.

(legend continued on next page)

In contrast to previous studies, rather than using a targeted approach to follow up only a small number of potential Vpr targets, we combined complementary proteomics analyses to describe the global proteome remodeling caused by Vpr and multiple direct substrates for Vpr-mediated depletion. The two established criteria for Vpr targets (binding and destabilization) are satisfied by numerous proteins identified here, including ESCO2, SMN1, BBX, and KIF18A. These proteins are therefore not candidate Vpr targets, but *bona fide* Vpr targets, proven to the same standard of evidence as other, previously described substrates.

However, in our proposed model, Vpr binds and degrades multiple cellular proteins, with the total pool of Vpr shared over multiple targets. Therefore, identification of cellular targets by coIP is technically problematic and more prone to false negatives compared with other proteins that establish interactions with a small number of binding partners. We therefore used an alternative method of identifying direct targets for Vpr-mediated depletion: proteins that are post-translationally degraded within 6 h of treatment with Vpr. We are confident that the other proteins identified in this fashion also represent direct targets for Vpr-mediated degradation because secondary effects are excluded by both intrinsic elements of the technique and the short time frame allowed.

Despite the importance of Vpr *in vivo*, a positive *in vitro* viral replication phenotype is often absent in T cell infection models (Goh et al., 1998; Guenzel et al., 2014). Nonetheless, expression of Vpr in T cells causes cell cycle arrest (Bolton and Lenardo, 2007; Gummuluru and Emerman, 1999; Rogel et al., 1995); cell death (Bolton and Lenardo, 2007); transactivation of the viral LTR (Gummuluru and Emerman, 1999); enhancement or antagonism of crucial signaling pathways, including NFAT (Höhne et al., 2016) and NF-κB (Liang et al., 2015; Liu et al., 2014; Muthumani et al., 2006); disruption of PARP1 localization (Höhne et al., 2016; Muthumani et al., 2006); defects in chromatid cohesion (Shimura et al., 2011); and induction of the DNA damage response (Richard et al., 2010; Vassena et al., 2013). At least some of these phenotypes can be segregated (Bolton and Lenardo, 2007; Höhne et al., 2016). The molecular mechanisms underpinning these phenomena have remained controversial. In our model, these multiple phenotypes can be explained by Vpr targeting multiple cellular proteins and pathways with potential for redundant or cumulative effects.

Here we considered the most well-described cellular phenotype for Vpr, G2/M cell cycle arrest, with findings compatible with this model. In addition to confirming the previously described effect of Vpr-mediated MCM10 degradation, we identified three other proteins that are directly targeted by Vpr, show depletion correlating with the extent of G2/M-mediated arrest in a panel of Vpr mutants, and result in arrest at G2/M when depleted through RNAi. Notably, depletion of two of these proteins, SMN1 and CDCA2 (Repo-Man), has been shown to activate the DNA damage response and stimulate ATM and/or ATR kinase activity (Kannan et al., 2018; Peng et al., 2010), a critical step toward G2/M arrest caused by Vpr (Berger et al., 2015; Fregoso and Emerman, 2016; Roshal et al., 2003). The contribution of multiple Vpr targets to the same cellular phenotype may also be exemplified by another described cellular phenotype for Vpr, premature chromatid segregation (PCS) (Shimura et al., 2011). Although not specifically investigated here, RNAi-mediated knockdown of three proteins depleted by Vpr—ESCO2, CDCA5 (Sororin), and HASPIN—has been associated previously with this phenotype in different systems (Dai et al., 2006; Hou and Zou, 2005; Rankin et al., 2005).

In conclusion, Vpr degrades multiple cellular targets, resulting in global remodeling of the host proteome and labyrinthine changes to different cellular pathways. This explains why its effects on cellular phenotypes and viral replication are complex and remain poorly understood, why the functional consequences of individual Vpr targets identified and studied in isolation have proved elusive, and why the search for a single critical Vpr target has been problematic.

STAR★METHODS

Detailed methods are provided in the online version of this paper and include the following:

- KEY RESOURCES TABLE
- CONTACT FOR REAGENT AND RESOURCE SHARING
- EXPERIMENTAL MODEL AND SUBJECT DETAILS
  - Cell lines
- METHOD DETAILS
  - NL4-3 molecular clones
  - Viral stocks
  - Lentivector for shRNA expression
  - CEM-T4 T cell infections
  - Immunoblotting
  - ShRNA knockdown confirmation by RT-PCR
  - 7-AAD staining
  - Immunoprecipitation Mass spectrometry (IP-MS)
  - Labeling with amino acids in cell culture (Pulsed-SILAC)
  - J-LAT reactivation experiments
  - Sample Preparation for Mass spectrometry
  - Offline high pH reversed-phase (HpRP) peptide fractionation
  - Mass spectrometry
- QUANTIFICATION AND STATISTICAL ANALYSIS
  - Gene ontology enrichment
  - MS Data processing and analysis
  - Phylogenetic tree of Vpr sequences
- DATA AND SOFTWARE ANALYSIS

SUPPLEMENTAL INFORMATION

Supplemental Information can be found online at https://doi.org/10.1016/j.celrep.2019.04.025.

(E) Graphical summary of the TMT experiment to examine proteome changes in cells transduced with HIV-2 Vpr and Vpx for an extended period.

(F) Scatterplots displaying pairwise comparison between cells transduced with 7312a HIV-2 Vpr and Vpx for 96 h compared with those transduced with an empty vector for 96 h. Blue and red dots represent the defined groups or proteins depleted or increased by NL4-3 Vpr, respectively. Points ringed in gold indicate the direct targets of NL4-3 Vpr-mediated degradation listed in Table 1.

OPEN
ACCESS
CellPress

ACKNOWLEDGMENTS

This work was supported by the Wellcome Trust (PRF 210688/Z/18/Z to P.J.L.), the MRC (CSF MR/P008801/1 to N.J.M.), NHSBT (WPA15-02 to N.J.M.), the NIHR Cambridge BRC, and a Wellcome Trust strategic award to the CIMR. The authors thank Dr. Reiner Schulte and the CIMR Flow Cytometry Core Facility team and the Lehner laboratory for critical discussions.

AUTHOR CONTRIBUTIONS

E.J.D.G., J.C.W., N.J.M., and P.J.L. conceived and planned the experiments. E.J.D.G., J.C.W., A.S., and A.N. carried out the experiments. E.J.D.G., J.C.W., N.J.M., and P.J.L. wrote the manuscript with input from all authors.

DECLARATION OF INTERESTS

The authors declare no competing interests.

Received: January 9, 2019
Revised: March 19, 2019
Accepted: April 2, 2019
Published: April 30, 2019

REFERENCES

Ashburner, M., Ball, C.A., Blake, J.A., Botstein, D., Butler, H., Cherry, J.M., Davis, A.P., Dolinski, K., Dwight, S.S., Eppig, J.T., et al.; The Gene Ontology Consortium (2000). Gene ontology: tool for the unification of biology. Nat. Genet. *25*, 25–29.

Ayyavoo, V., Mahboubi, A., Mahalingam, S., Ramalingam, R., Kudchodkar, S., Williams, W.V., Green, D.R., and Weiner, D.B. (1997). HIV-1 Vpr suppresses immune activation and apoptosis through regulation of nuclear factor kappa B. Nat. Med. *3*, 1117–1123.

Belzile, J.P., Richard, J., Rougeau, N., Xiao, Y., and Cohen, E.A. (2010). HIV-1 Vpr induces the K48-linked polyubiquitination and proteasomal degradation of target cellular proteins to activate ATR and promote G2 arrest. J. Virol. *84*, 3320–3330.

Berger, G., Lawrence, M., Hué, S., and Neil, S.J. (2015). G2/M cell cycle arrest correlates with primate lentiviral Vpr interaction with the SLX4 complex. J. Virol. *89*, 230–240.

Boisvert, F.M., Ahmad, Y., Gierlinski, M., Charriere, F., Lamont, D., Scott, M., Barton, G., and Lamond, A.I. (2012). A quantitative spatial proteomics analysis of proteome turnover in human cells. Mol. Cell Proteomics *11*, M111.011429.

Bolton, D.L., and Lenardo, M.J. (2007). Vpr cytopathicity independent of G2/M cell cycle arrest in human immunodeficiency virus type 1-infected CD4+ T cells. J. Virol. *81*, 8878–8890.

Cano, F., Rapiteanu, R., Sebastiaan Winkler, G., and Lehner, P.J. (2015). A non-proteolytic role for ubiquitin in deadenylation of MHC-I mRNA by the RNA-binding E3-ligase MEX-3C. Nat. Commun. *6*, 8670.

Chougui, G., Munir-Matloob, S., Matkovic, R., Martin, M.M., Morel, M., Lahouassa, H., Leduc, M., Ramirez, B.C., Etienne, L., and Margottin-Goguet, F. (2018). HIV-2/SIV viral protein X counteracts HUSH repressor complex. Nat. Microbiol. *3*, 891–897.

Connor, R.I., Chen, B.K., Choe, S., and Landau, N.R. (1995). Vpr is required for efficient replication of human immunodeficiency virus type-1 in mononuclear phagocytes. Virology *206*, 935–944.

Cox, J., and Mann, M. (2008). MaxQuant enables high peptide identification rates, individualized p.p.b.-range mass accuracies and proteome-wide protein quantification. Nat. Biotechnol. *26*, 1367–1372.

Coyaud, E., Ranadheera, C., Cheng, D., Gonçalves, J., Dyakov, B.J.A., Laurent, E.M.N., St-Germain, J., Pelletier, L., Gingras, A.C., Brumell, J.H., et al. (2018). Global Interactomics Uncovers Extensive Organellar Targeting by Zika Virus. Mol. Cell. Proteomics *17*, 2242–2255.

Dai, J., Sullivan, B.A., and Higgins, J.M. (2006). Regulation of mitotic chromosome cohesion by Haspin and Aurora B. Dev. Cell *11*, 741–750.

Dehart, J.L., and Planelles, V. (2008). Human immunodeficiency virus type 1 Vpr links proteasomal degradation and checkpoint activation. J. Virol. *82*, 1066–1072.

Diamond, D.L., Syder, A.J., Jacobs, J.M., Sorensen, C.M., Walters, K.A., Proll, S.C., McDermott, J.E., Gritsenko, M.A., Zhang, Q., Zhao, R., et al. (2010). Temporal proteome and lipidome profiles reveal hepatitis C virus-associated reprogramming of hepatocellular metabolism and bioenergetics. PLoS Pathog. *6*, e1000719.

Ersing, I., Nobre, L., Wang, L.W., Soday, L., Ma, Y., Paulo, J.A., Narita, Y., Ashbaugh, C.W., Jiang, C., Grayson, N.E., et al. (2017). A Temporal Proteomic Map of Epstein-Barr Virus Lytic Replication in B Cells. Cell Rep. *19*, 1479–1493.

Felzien, L.K., Woffendin, C., Hottiger, M.O., Subbramanian, R.A., Cohen, E.A., and Nabel, G.J. (1998). HIV transcriptional activation by the accessory protein, VPR, is mediated by the p300 co-activator. Proc. Natl. Acad. Sci. USA *95*, 5281–5286.

Fischer, M., Grossmann, P., Padi, M., and DeCaprio, J.A. (2016). Integration of TP53, DREAM, MMB-FOXM1 and RB-E2F target gene analyses identifies cell cycle gene regulatory networks. Nucleic Acids Res. *44*, 6070–6086.

Fletcher, T.M., 3rd, Brichacek, B., Sharova, N., Newman, M.A., Stivahtis, G., Sharp, P.M., Emerman, M., Hahn, B.H., and Stevenson, M. (1996). Nuclear import and cell cycle arrest functions of the HIV-1 Vpr protein are encoded by two separate genes in HIV-2/SIV(SM). EMBO J. *15*, 6155–6165.

Foley, G.E., Lazarus, H., Farber, S., Uzman, B.G., Boone, B.A., and McCarthy, R.E. (1965). Continuous Culture of Human Lymphoblasts from Peripheral Blood of a Child with Acute Leukemia. Cancer *18*, 522–529.

Forget, J., Yao, X.J., Mercier, J., and Cohen, E.A. (1998). Human immunodeficiency virus type 1 vpr protein transactivation function: mechanism and identification of domains involved. J. Mol. Biol. *284*, 915–923.

Fregoso, O.I., and Emerman, M. (2016). Activation of the DNA Damage Response Is a Conserved Function of HIV-1 and HIV-2 Vpr That Is Independent of SLX4 Recruitment. MBio *7*, e01433-16.

Goh, W.C., Rogel, M.E., Kinsey, C.M., Michael, S.F., Fultz, P.N., Nowak, M.A., Hahn, B.H., and Emerman, M. (1998). HIV-1 Vpr increases viral expression by manipulation of the cell cycle: a mechanism for selection of Vpr in vivo. Nat. Med. *4*, 65–71.

Greenwood, E.J., Matheson, N.J., Wals, K., van den Boomen, D.J., Antrobus, R., Williamson, J.C., and Lehner, P.J. (2016). Temporal proteomic analysis of HIV infection reveals remodelling of the host phosphoproteome by lentiviral Vif variants. eLife *5*, e18296.

Guenzel, C.A., Hérate, C., and Benichou, S. (2014). HIV-1 Vpr-a still "enigmatic multitasker". Front. Microbiol. *5*, 127.

Gummuluru, S., and Emerman, M. (1999). Cell cycle- and Vpr-mediated regulation of human immunodeficiency virus type 1 expression in primary and transformed T-cell lines. J. Virol. *73*, 5422–5430.

Guo, Z., Kong, Q., Liu, C., Zhang, S., Zou, L., Yan, F., Whitmire, J.K., Xiong, Y., Chen, X., and Wan, Y.Y. (2016). DCAF1 controls T-cell function via p53-dependent and -independent mechanisms. Nat. Commun. *7*, 10307.

Hofmann, S., Dehn, S., Businger, R., Bolduan, S., Schneider, M., Debyser, Z., Brack-Werner, R., and Schindler, M. (2017). Dual role of the chromatin-binding factor PHF13 in the pre- and post-integration phases of HIV-1 replication. Open Biol. *7*, 170115.

Höhne, K., Businger, R., van Nuffel, A., Bolduan, S., Koppensteiner, H., Baeyens, A., Vermeire, J., Malatinkova, E., Verhasselt, B., and Schindler, M. (2016). Virion encapsidated HIV-1 Vpr induces NFAT to prime non-activated T cells for productive infection. Open Biol. *6*, 160046.

Hossain, D., Javadi Esfehani, Y., Das, A., and Tsang, W.Y. (2017). Cep78 controls centrosome homeostasis by inhibiting EDD-DYRK2-DDB1^{VprBP}. EMBO Rep. *18*, 632–644.

Hou, F., and Zou, H. (2005). Two human orthologues of Eco1/Ctf7 acetyltransferases are both required for proper sister-chromatid cohesion. Mol. Biol. Cell *16*, 3908–3918.

Hrecka, K., Hao, C., Gierszewska, M., Swanson, S.K., Kesik-Brodacka, M., Srivastava, S., Florens, L., Washburn, M.P., and Skowronski, J. (2011). Vpx relieves inhibition of HIV-1 infection of macrophages mediated by the SAMHD1 protein. Nature *474*, 658–661.

Hrecka, K., Hao, C., Shun, M.C., Kaur, S., Swanson, S.K., Florens, L., Washburn, M.P., and Skowronski, J. (2016). HIV-1 and HIV-2 exhibit divergent interactions with HLTF and UNG2 DNA repair proteins. Proc. Natl. Acad. Sci. USA *113*, E3921–E3930.

Huber, W., Carey, V.J., Gentleman, R., Anders, S., Carlson, M., Carvalho, B.S., Bravo, H.C., Davis, S., Gatto, L., Girke, T., et al. (2015). Orchestrating high-throughput genomic analysis with Bioconductor. Nat. Methods *12*, 115–121.

Hughes, C.S., Foehr, S., Garfield, D.A., Furlong, E.E., Steinmetz, L.M., and Krijgsveld, J. (2014). Ultrasensitive proteome analysis using paramagnetic bead technology. Mol. Syst. Biol. *10*, 757.

Jaffar, S., Grant, A.D., Whitworth, J., Smith, P.G., and Whittle, H. (2004). The natural history of HIV-1 and HIV-2 infections in adults in Africa: a literature review. Bull. World Health Organ. *82*, 462–469.

Jäger, S., Cimermancic, P., Gulbahce, N., Johnson, J.R., McGovern, K.E., Clarke, S.C., Shales, M., Mercenne, G., Pache, L., Li, K., et al. (2011). Global landscape of HIV-human protein complexes. Nature *481*, 365–370.

Jordan, A., Defechereux, P., and Verdin, E. (2001). The site of HIV-1 integration in the human genome determines basal transcriptional activity and response to Tat transactivation. EMBO J. *20*, 1726–1738.

Jordan, A., Bisgrove, D., and Verdin, E. (2003). HIV reproducibly establishes a latent infection after acute infection of T cells in vitro. EMBO J. *22*, 1868–1877.

Kannan, A., Bhatia, K., Branzei, D., and Gangwani, L. (2018). Combined deficiency of Senataxin and DNA-PKcs causes DNA damage accumulation and neurodegeneration in spinal muscular atrophy. Nucleic Acids Res. *46*, 8326–8346.

Laguette, N., Sobhian, B., Casartelli, N., Ringeard, M., Chable-Bessia, C., Ségéral, E., Yatim, A., Emiliani, S., Schwartz, O., and Benkirane, M. (2011). SAMHD1 is the dendritic- and myeloid-cell-specific HIV-1 restriction factor counteracted by Vpx. Nature *474*, 654–657.

Laguette, N., Brégnard, C., Hue, P., Basbous, J., Yatim, A., Larroque, M., Kirchhoff, F., Constantinou, A., Sobhian, B., and Benkirane, M. (2014). Premature activation of the SLX4 complex by Vpr promotes G2/M arrest and escape from innate immune sensing. Cell *156*, 134–145.

Lahouassa, H., Blondot, M.L., Chauveau, L., Chougui, G., Morel, M., Leduc, M., Guillonneau, F., Ramirez, B.C., Schwartz, O., and Margottin-Goguet, F. (2016). HIV-1 Vpr degrades the HLTF DNA translocase in T cells and macrophages. Proc. Natl. Acad. Sci. U.S.A *113*, 5311–5316.

Lahti, A.L., Manninen, A., and Saksela, K. (2003). Regulation of T cell activation by HIV-1 accessory proteins: Vpr acts via distinct mechanisms to cooperate with Nef in NFAT-directed gene expression and to promote transactivation by CREB. Virology *310*, 190–196.

Lapek, J.D., Jr., Lewinski, M.K., Wozniak, J.M., Guatelli, J., and Gonzalez, D.J. (2017). Quantitative Temporal Viromics of an Inducible HIV-1 Model Yields Insight to Global Host Targets and Phospho-Dynamics Associated with Protein Vpr. Mol. Cell. Proteomics *16*, 1447–1461.

Le Rouzic, E., Belaïdouni, N., Estrabaud, E., Morel, M., Rain, J.C., Transy, C., and Margottin-Goguet, F. (2007). HIV1 Vpr arrests the cell cycle by recruiting DCAF1/VprBP, a receptor of the Cul4-DDB1 ubiquitin ligase. Cell Cycle *6*, 182–188.

León, I.R., Schwämmle, V., Jensen, O.N., and Sprenger, R.R. (2013). Quantitative assessment of in-solution digestion efficiency identifies optimal protocols for unbiased protein analysis. Mol. Cell. Proteomics *12*, 2992–3005.

Liang, Z., Liu, R., Lin, Y., Liang, C., Tan, J., and Qiao, W. (2015). HIV-1 Vpr protein activates the NF-κB pathway to promote G2/M cell cycle arrest. Virol. Sin. *30*, 441–448.

Lim, E.S., Fregoso, O.I., McCoy, C.O., Matsen, F.A., Malik, H.S., and Emerman, M. (2012). The ability of primate lentiviruses to degrade the monocyte restriction factor SAMHD1 preceded the birth of the viral accessory protein Vpx. Cell Host Microbe *11*, 194–204.

Liu, R., Lin, Y., Jia, R., Geng, Y., Liang, C., Tan, J., and Qiao, W. (2014). HIV-1 Vpr stimulates NF-κB and AP-1 signaling by activating TAK1. Retrovirology *11*, 45.

Luo, Y., Jacobs, E.Y., Greco, T.M., Mohammed, K.D., Tong, T., Keegan, S., Binley, J.M., Cristea, I.M., Fenyö, D., Rout, M.P., et al. (2016). HIV-host interactome revealed directly from infected cells. Nat. Microbiol. *1*, 16068.

Lv, L., Wang, Q., Xu, Y., Tsao, L.C., Nakagawa, T., Guo, H., Su, L., and Xiong, Y. (2018). Vpr Targets TET2 for Degradation by CRL4(VprBP) E3 Ligase to Sustain IL-6 Expression and Enhance HIV-1 Replication. Mol. Cell *70*, 961–970.e5.

Ly, T., Endo, A., and Lamond, A.I. (2015). Proteomic analysis of the response to cell cycle arrests in human myeloid leukemia cells. eLife *4*.

Mashiba, M., Collins, D.R., Terry, V.H., and Collins, K.L. (2015). Vpr Overcomes Macrophage-Specific Restriction of HIV-1 Env Expression and Virion Production. Cell Host Microbe *17*, 414.

Matheson, N.J., Peden, A.A., and Lehner, P.J. (2014). Antibody-free magnetic cell sorting of genetically modified primary human CD4+ T cells by one-step streptavidin affinity purification. PLoS ONE *9*, e111437.

Matheson, N.J., Sumner, J., Wals, K., Rapiteanu, R., Weekes, M.P., Vigan, R., Weinelt, J., Schindler, M., Antrobus, R., Costa, A.S., et al. (2015). Cell Surface Proteomic Map of HIV Infection Reveals Antagonism of Amino Acid Metabolism by Vpu and Nef. Cell Host Microbe *18*, 409–423.

Matheson, N.J., Greenwood, E.J., and Lehner, P.J. (2016). Manipulation of immunometabolism by HIV-accessories to the crime? Curr. Opin. Virol. *19*, 65–70.

Maudet, C., Sourisce, A., Dragin, L., Lahouassa, H., Rain, J.C., Bouaziz, S., Ramirez, B.C., and Margottin-Goguet, F. (2013). HIV-1 Vpr induces the degradation of ZIP and sZIP, adaptors of the NuRD chromatin remodeling complex, by hijacking DCAF1/VprBP. PLoS ONE *8*, e77320.

Mellacheruvu, D., Wright, Z., Couzens, A.L., Lambert, J.P., St-Denis, N.A., Li, T., Miteva, Y.V., Hauri, S., Sardiu, M.E., Low, T.Y., et al. (2013). The CRAPome: a contaminant repository for affinity purification-mass spectrometry data. Nat. Methods *10*, 730–736.

Mi, H., Huang, X., Muruganujan, A., Tang, H., Mills, C., Kang, D., and Thomas, P.D. (2017). PANTHER version 11: expanded annotation data from Gene Ontology and Reactome pathways, and data analysis tool enhancements. Nucleic Acids Res. *45* (*D1*), D183–D189.

Miller, C.M., Akiyama, H., Agosto, L.M., Emery, A., Ettinger, C.R., Swanstrom, R.I., Henderson, A.J., and Gummuluru, S. (2017). Virion-Associated Vpr Alleviates a Postintegration Block to HIV-1 Infection of Dendritic Cells. J. Virol. *91*, e00051-17.

Murray, J.M., Kelleher, A.D., and Cooper, D.A. (2011). Timing of the components of the HIV life cycle in productively infected CD4+ T cells in a population of HIV-infected individuals. J. Virol. *85*, 10798–10805.

Muthumani, K., Choo, A.Y., Zong, W.X., Madesh, M., Hwang, D.S., Premkumar, A., Thieu, K.P., Emmanuel, J., Kumar, S., Thompson, C.B., and Weiner, D.B. (2006). The HIV-1 Vpr and glucocorticoid receptor complex is a gain-of-function interaction that prevents the nuclear localization of PARP-1. Nat. Cell Biol. *8*, 170–179.

Naamati, A., Williamson, J.C., Greenwood, E.J., Marelli, S., Lehner, P.J., and Matheson, N.J. (2019). Functional proteomic atlas of HIV infection in primary human CD4+ T cells. eLife *8*, e41431.

Peng, A., Lewellyn, A.L., Schiemann, W.P., and Maller, J.L. (2010). Repo-man controls a protein phosphatase 1-dependent threshold for DNA damage checkpoint activation. Curr. Biol. *20*, 387–396.

Perelson, A.S., Neumann, A.U., Markowitz, M., Leonard, J.M., and Ho, D.D. (1996). HIV-1 dynamics in vivo: virion clearance rate, infected cell life-span, and viral generation time. Science *271*, 1582–1586.

Poon, B., Grovit-Ferbas, K., Stewart, S.A., and Chen, I.S. (1998). Cell cycle arrest by Vpr in HIV-1 virions and insensitivity to antiretroviral agents. Science *281*, 266–269.

R Core Team (2013). R: A language and environment for statistical computing (R Foundation for Statistical Computing).

Rankin, S., Ayad, N.G., and Kirschner, M.W. (2005). Sororin, a substrate of the anaphase-promoting complex, is required for sister chromatid cohesion in vertebrates. Mol. Cell *18*, 185–200.

Re, F., Braaten, D., Franke, E.K., and Luban, J. (1995). Human immunodeficiency virus type 1 Vpr arrests the cell cycle in G2 by inhibiting the activation of p34cdc2-cyclin B. J. Virol. *69*, 6859–6864.

Richard, J., Sindhu, S., Pham, T.N., Belzile, J.P., and Cohen, E.A. (2010). HIV-1 Vpr up-regulates expression of ligands for the activating NKG2D receptor and promotes NK cell-mediated killing. Blood *115*, 1354–1363.

Rogel, M.E., Wu, L.I., and Emerman, M. (1995). The human immunodeficiency virus type 1 vpr gene prevents cell proliferation during chronic infection. J. Virol. *69*, 882–888.

Romani, B., Shaykh Baygloo, N., Aghasadeghi, M.R., and Allahbakhshi, E. (2015). HIV-1 Vpr Protein Enhances Proteasomal Degradation of MCM10 DNA Replication Factor through the Cul4-DDB1[VprBP] E3 Ubiquitin Ligase to Induce G2/M Cell Cycle Arrest. J. Biol. Chem. *290*, 17380–17389.

Roshal, M., Kim, B., Zhu, Y., Nghiem, P., and Planelles, V. (2003). Activation of the ATR-mediated DNA damage response by the HIV-1 viral protein R. J. Biol. Chem. *278*, 25879–25886.

Roux, P., Alfieri, C., Hrimech, M., Cohen, E.A., and Tanner, J.E. (2000). Activation of transcription factors NF-kappaB and NF-IL-6 by human immunodeficiency virus type 1 protein R (Vpr) induces interleukin-8 expression. J. Virol. *74*, 4658–4665.

Schröfelbauer, B., Yu, Q., Zeitlin, S.G., and Landau, N.R. (2005). Human immunodeficiency virus type 1 Vpr induces the degradation of the UNG and SMUG uracil-DNA glycosylases. J. Virol. *79*, 10978–10987.

Schwämmle, V., León, I.R., and Jensen, O.N. (2013). Assessment and improvement of statistical tools for comparative proteomics analysis of sparse data sets with few experimental replicates. J. Proteome Res. *12*, 3874–3883.

Selig, L., Benichou, S., Rogel, M.E., Wu, L.I., Vodicka, M.A., Sire, J., Benarous, R., and Emerman, M. (1997). Uracil DNA glycosylase specifically interacts with Vpr of both human immunodeficiency virus type 1 and simian immunodeficiency virus of sooty mangabeys, but binding does not correlate with cell cycle arrest. J. Virol. *71*, 4842–4846.

Shimura, M., Toyoda, Y., Iijima, K., Kinomoto, M., Tokunaga, K., Yoda, K., Yanagida, M., Sata, T., and Ishizaka, Y. (2011). Epigenetic displacement of HP1 from heterochromatin by HIV-1 Vpr causes premature sister chromatid separation. J. Cell Biol. *194*, 721–735.

Simon, V., Bloch, N., and Landau, N.R. (2015). Intrinsic host restrictions to HIV-1 and mechanisms of viral escape. Nat. Immunol. *16*, 546–553.

Sugden, S.M., Bego, M.G., Pham, T.N., and Cohen, E.A. (2016). Remodeling of the Host Cell Plasma Membrane by HIV-1 Nef and Vpu: A Strategy to Ensure Viral Fitness and Persistence. Viruses *8*, 67.

Sumner, R.P., Thorne, L.G., Fink, D.L., Khan, H., Milne, R.S., and Towers, G.J. (2017). Are Evolution and the Intracellular Innate Immune System Key Determinants in HIV Transmission? Front. Immunol. *8*, 1246.

Tchasovnikarova, I.A., Timms, R.T., Matheson, N.J., Wals, K., Antrobus, R., Göttgens, B., Dougan, G., Dawson, M.A., and Lehner, P.J. (2015). GENE SILENCING. Epigenetic silencing by the HUSH complex mediates position-effect variegation in human cells. Science *348*, 1481–1485.

Terada, Y., and Yasuda, Y. (2006). Human immunodeficiency virus type 1 Vpr induces G2 checkpoint activation by interacting with the splicing factor SAP145. Mol. Cell. Biol. *26*, 8149–8158.

The Gene Ontology Consortium (2019). The Gene Ontology Resource: 20 years and still GOing strong. Nucleic Acids Res. *47* (*D1*), D330–D338.

Tyanova, S., Temu, T., Sinitcyn, P., Carlson, A., Hein, M.Y., Geiger, T., Mann, M., and Cox, J. (2016). The Perseus computational platform for comprehensive analysis of (prote)omics data. Nat. Methods *13*, 731–740.

UniProt Consortium (2019). UniProt: a worldwide hub of protein knowledge. Nucleic Acids Res. *47* (*D1*), D506–D515.

van den Boomen, D.J., Timms, R.T., Grice, G.L., Stagg, H.R., Skødt, K., Dougan, G., Nathan, J.A., and Lehner, P.J. (2014). TMEM129 is a Derlin-1 associated ERAD E3 ligase essential for virus-induced degradation of MHC-I. Proc. Natl. Acad. Sci. USA *111*, 11425–11430.

Vassena, L., Giuliani, E., Matusali, G., Cohen, E.A., and Doria, M. (2013). The human immunodeficiency virus type 1 Vpr protein upregulates PVR via activation of the ATR-mediated DNA damage response pathway. J. Gen. Virol. *94*, 2664–2669.

Vizcaíno, J.A., Csordas, A., Del-Toro, N., Dianes, J.A., Griss, J., Lavidas, I., Mayer, G., Perez-Riverol, Y., Reisinger, F., Ternent, T., et al. (2016). 2016 update of the PRIDE database and its related tools. Nucleic Acids Res. *44*, 11033.

Waterhouse, A.M., Procter, J.B., Martin, D.M., Clamp, M., and Barton, G.J. (2009). Jalview Version 2—a multiple sequence alignment editor and analysis workbench. Bioinformatics *25*, 1189–1191.

Weekes, M.P., Tomasec, P., Huttlin, E.L., Fielding, C.A., Nusinow, D., Stanton, R.J., Wang, E.C., Aicheler, R., Murrell, I., Wilkinson, G.W., et al. (2014). Quantitative temporal viromics: an approach to investigate host-pathogen interaction. Cell *157*, 1460–1472.

Wu, Y., Zhou, X., Barnes, C.O., DeLucia, M., Cohen, A.E., Gronenborn, A.M., Ahn, J., and Calero, G. (2016). The DDB1-DCAF1-Vpr-UNG2 crystal structure reveals how HIV-1 Vpr steers human UNG2 toward destruction. Nat. Struct. Mol. Biol. *23*, 933–940.

Yurkovetskiy, L., Guney, M.H., Kim, K., Goh, S.L., McCauley, S., Dauphin, A., Diehl, W.E., and Luban, J. (2018). Primate immunodeficiency virus proteins Vpx and Vpr counteract transcriptional repression of proviruses by the HUSH complex. Nat. Microbiol. *3*, 1354–1361.

Zhang, H., Zhou, Y., Alcock, C., Kiefer, T., Monie, D., Siliciano, J., Li, Q., Pham, P., Cofrancesco, J., Persaud, D., and Siliciano, R.F. (2004). Novel single-cell-level phenotypic assay for residual drug susceptibility and reduced replication capacity of drug-resistant human immunodeficiency virus type 1. J. Virol. *78*, 1718–1729.

Zhou, Y., and Ratner, L. (2000). Phosphorylation of human immunodeficiency virus type 1 Vpr regulates cell cycle arrest. J. Virol. *74*, 6520–6527.

Zhou, X., DeLucia, M., and Ahn, J. (2016). SLX4-SLX1 Protein-independent Down-regulation of MUS81-EME1 Protein by HIV-1 Viral Protein R (Vpr). J. Biol. Chem. *291*, 16936–16947.

Zufferey, R., Nagy, D., Mandel, R.J., Naldini, L., and Trono, D. (1997). Multiply attenuated lentiviral vector achieves efficient gene delivery in vivo. Nat. Biotechnol. *15*, 871–875.

STAR★METHODS

KEY RESOURCES TABLE

REAGENT or RESOURCE	SOURCE	IDENTIFIER
Antibodies		
Rabbit anti-TASOR	Atlas Antibodies	Cat#HPA006735; RRID: AB_1852384
Rabbit anti-BBX	Bethyl Laboratories	Cat#A303-151A; RRID: AB_10893371
Rabbit anti-HLTF	Bethyl Laboratories	Cat#A300-230A; RRID: AB_2117307
Rabbit anti-RALY	Bethyl Laboratories	Cat#A302-070A; RRID: AB_1604220
Rabbit anti-ZNF512B	Bethyl Laboratories	Cat#A303-234A; RRID: AB_10952552
Mouse anti-SMN1/2	Cell Signaling Technology	Cat#12976S; RRID: AB_2798076
Rabbit anti-ESCO2	Novus Biologicals	Cat#NB100-87021; RRID: AB_1201179
Mouse anti-UNG2	Origene	Cat#TA503563; RRID: AB_11126624
Rabbit anti-Vpr	Proteintech	Cat#51143-I-AP;RRID: AB_10695191
Mouse anti-CCNB1	Santa Cruz	Cat#SC-245; RRID: AB_627338
Mouse anti-ZGPAT	Santa Cruz	Cat#SC-515524
Mouse anti-β-actin	Sigma-Aldrich	Cat#A5316; RRID: AB_476743
Mouse anti-p24	Abcam	Cat#ab9071; RRID: AB_306981
Mouse anti-VCP	Abcam	Cat#ab11433; RRID: AB_298039
Goat anti-mouse HRP	Jackson ImmunoResearch	Cat#115-035-146; RRID: AB_2307392
Goat anti-rabbit HRP	Jackson ImmunoResearch	Cat#111-035-144; RRID: AB_2307391
Mouse anti-CD4-AF647	Biolegend	Cat#317422; RRID: AB_571941
Mouse anti-CD271(NGFR)-APC	Biolegend	Cat#345107; RRID: AB_10639737
Chemicals, Peptides, and Recombinant Proteins		
Sigma EZview Red Anti-HA Affinity Gel	Sigma-Aldrich	Cat#E6779
IgG-Sepharose	GE Healthcare	Cat#17096901
IGEPAL CA-630 (NP-40)	Sigma-Aldrich	Cat#I3021
Benzonase	Sigma-Aldrich	Cat#E1014
MLN4924	Millipore	Cat#5054770001
Zidovudine (AZT)	NIH AIDS Reagent Program	Cat#3485
Efavirenz	NIH AIDS Reagent Program	Cat#4624
7-AAD	Stratech	Cat#17501-AAT
TNFα	PeproTech, 300-01A	Cat#300-01A
R10 Arginine	Cambridge Isotope Laboratories	Cat#CNLM-539
R6 Arginine	Cambridge Isotope Laboratories	Cat#CLM-2265
K8 Lysine	Cambridge Isotope Laboratories	Cat#CNLM-291
K4 Lysine	Cambridge Isotope Laboratories	Cat#DLM-2640
TMT10plex Isobaric Label Reagent	Thermo Fisher Scientific	Cat#90110
TMT11-131C Isobaric Label Reagent	Thermo Fisher Scientific	Cat#A34807
PreOmics-IST NHS Sample preparation kit	PreOmics	Cat#P.O.00030
SpeedBead Carboxylate modified magnetic particles	GE Healthcare	Cat#45152105050250
SpeedBead Carboxylate modified magnetic particles	GE Healthcare	Cat#65152105050250
Trypsin, Mass Spectrometry Grade	Thermo Fisher Scientific	Cat#90057
Deposited Data		
Raw proteomics data	This paper	PRIDE: PXD013221
UniProt Human reference proteome (26/09/2017)	Uniprot (UniProt Consortium, 2019)	https://www.uniprot.org
trEMBL (Human) sequence database (26/09/2017)	Uniprot (UniProt Consortium, 2019)	https://www.uniprot.org

(Continued on next page)

Continued

REAGENT or RESOURCE	SOURCE	IDENTIFIER
GO Ontology database (06/09/2018)	Gene Ontology Consortium (The Gene Ontology Consortium, 2019)	Accessed through http://www.pantherdb.org
CRAPome v1.1	(Mellacheruvu et al., 2013)	http://www.crapome.org/
Experimental Models: Cell Lines		
CEM-T4	NIH AIDS Reagent Program, Dr JP Jacobs (Foley et al., 1965),	Cat#117
J-Lat Tat-GFP Cells (A1)	NIH AIDS Reagent Program, Dr E Verdin (Jordan et al., 2003; Jordan et al., 2001)	Cat#9852
HEK293T	Lehner Lab stock	RRID:CVCL_0063
Oligonucleotides		
MCM10_FOR 5′-CTTATACAGAAGAGGCTGATG-3′	Sigma-Aldrich	KiCqStart: H_MCM10_1
MCM10_REV 5′-CCTCTTGCAACTCTTCATTC-3′	Sigma-Aldrich	KiCqStart: H_MCM10_1
ZNF267_FOR 5′-GTAGAATTCTCTTTGGAGGAG	Sigma-Aldrich	KiCqStart: H_ZNF267_1
ZNF267_REV 5′-CTCACTCTTCACATTCCAAG	Sigma-Aldrich	KiCqStart: H_ZNF267_1
CDCA2_FOR 5′-AGGAAAGTCATCATCCTACC	Sigma-Aldrich	KiCqStart: H_CDCA2_1
CDCA2_REV 5′-GATGGTTTGTTTCAGGAGAG-3	Sigma-Aldrich	KiCqStart: H_CDCA2_1
SMN1_FOR 5′-GGAAAGCCAGGTCTAAAATTC-3	Sigma-Aldrich	KiCqStart: H_SMN1_1
SMN1_REV 5′-AGAATCTGGACATATGGGAG-3	Sigma-Aldrich	KiCqStart: H_SMN1_1
GAPDH_FOR 5′ ATGGGGAAGGTGAAGGTCG-3	Cano et al., 2015	N/A
GAPDH_REV 5- CTCCACGACGTACTCAGCG-3	Cano et al., 2015	N/A
Recombinant DNA		
pNL4-3-dE-EGFP	NIH AIDS Reagent Program, Drs Haili Zhang, Yan Zhou, and Robert Siliciano (Zhang et al., 2004)	Cat#11100
pNL4-3-dE-EGFP-dVpr	This paper	N/A
pHRSIN RSV NL4.3 Vpr Ub Emerald	This paper	N/A
pHRSIN RSV NL4.3 Vpr S79A Ub Emerald	This paper	N/A
pHRSIN RSV NL4.3 Vpr Q65R Ub Emerald	This paper	N/A
pHRSIN RSV NL4.3 Vpr E24R R36P Ub Emerald	This paper	N/A
pHRSIN RSV NL4.3 Vpr W54R Ub Emerald	This paper	N/A
pHRSIN RSV NL4.3 Vpr Y47A, D52A, W54R Ub Emerald	This paper	N/A
pHRSIN RSV NL4.3 HA-Vpr Ub Emerald	This paper	N/A
pHRSIN RSV NL4.3 3xHA-Vpr Ub Emerald	This paper	N/A
pHRSIN RSV 98BR004 HA-Vpr Ub Emerald	This paper	N/A, but based on Vpr sequence GenBank: AAK31002.1
pHRSIN RSV BCF09 HA-Vpr Ub Emerald	This paper	N/A, but based on Vpr sequence GenBank: CAA75954.1
pHRSIN RSV SIVcpzPtt MB897 HA-Vpr Ub Emerald	This paper	N/A, but based on Vpr sequence GenBank: ABU53019.1
pHRSIN RSV SIVrcm 02CM8081 HA-Vpr Ub Emerald	This paper	N/A, but based on Vpr sequence GenBank: ADK78264.1
pHRSIN RSV SIVagm Sab92018 HA-Vpr Ub Emerald	This paper	N/A, but based on Vpr sequence GenBank: ADO34202.1
pHRSIN RSV SIVsmm E660 HA-Vpr Ub Emerald	This paper	N/A, but based on Vpr sequence GenBank: ANT86736.1
pHRSIN RSV HIV-2 ROD HA-Vpr Ub Emerald	This paper	N/A, but based on Vpr sequence GenBank: CAA28911.1
pHRSIN RSV HIV-2 7312a HA-Vpr Ub Emerald	This paper	N/A, but based on Vpr sequence GenBank: AAL31354.1

(*Continued on next page*)

Continued		
REAGENT or RESOURCE	SOURCE	IDENTIFIER
pHRSIN RSV SIVmus 01CM1239 HA-Vpr Ub Emerald	This paper	N/A, but based on Vpr sequence GenBank: ABO61047.1
pHRSIN RSV HIV-2 Rod HA-Vpx Ub Emerald	This paper	N/A, but based on Vpx sequence GenBank: AAB00766.1
pHRSIN RSV HIV-2 7312a HA-Vpx Ub Emerald	This paper	N/A, but based on Vpx sequence GenBank: AAL31353.1
pHRSIN RSV SIVrcm NG411 HA-Vpx Ub Emerald	This paper	N/A, but based on Vpx sequence GenBank: AAK69676.1
pHRSIN RSV SIVmnd-2 5440 HA-Vpx Ub Emerald	This paper	N/A, but based on Vpx sequence GenBank: AAO22477.1
pC.SIREN.puro shControl 1 (GTTATAGGCTCGCAAAAGG)	This paper	N/A
pC.SIREN.puro shControl 2 (GTAAGGCTATGAAGAGATAC)	This paper	N/A
pC.SIREN.puro shControl 3 (ACTACCGTTGTTATAGGTG)	This paper	N/A
pC.SIREN.hygro *DCAF1* (GCTGAGAATACTCTTCAAGAA)	This paper	N/A
pC.SIREN.puro sh*MCM10*-1 (AGATGCAGGAGCGCTACTTTG)	This paper	N/A
pC.SIREN.puro sh*MCM10*-2 (GACGGCGACGGTGAATCTTAT)	This paper	N/A
pC.SIREN.puro sh*CCNT1* (GCCTGCATTTGACCACATTTA)	This paper	N/A
pC.SIREN.puro sh*PINX1* (CCTTCAGCAAGAGAGTTTAAT)	This paper	N/A
pC.SIREN.puro sh*ZNF267*-1 (TACTCGTTCCTCCAATCTTAT)	This paper	N/A
pC.SIREN.puro sh*ZNF267*-2 (ATCAATATAGGAAGGTCTTTA)	This paper	N/A
pC.SIREN.puro sh*MBD1* (CACCAACCTTCCAAGTGTAAA)	This paper	N/A
pC.SIREN.puro sh*KIF20A* (CCGATGACGATGTCGTAGTTT)	This paper	N/A
pC.SIREN.puro sh*CWC25* (GATCACAGAAGAAGATGGCA)	This paper	N/A
pC.SIREN.puro sh*ZNF574* (ACCACCTTGTAAGTTCTAAAT)	This paper	N/A
pC.SIREN.puro sh*SMN1*-1 (ATCTGTGAAGTAGCTAATAA)	This paper	N/A
pC.SIREN.puro sh*SMN1*-2 (GGCTATCATACTGGCTATTAT)	This paper	N/A
pC.SIREN.puro sh*CDCA2*-1 (AGACTGGGTTCAGGTTATTTC)	This paper	N/A
pC.SIREN.puro shCDCA2-2 (CCGTTCTCAGTTCTCCTAATA)	This paper	N/A
pHRSIN NL4-3 Vpr IRES SBP-ΔLNGFR	This paper	N/A
pHRSIN HIV-2 Rod Vpx IRES SBP-ΔLNGFR	This paper	N/A
Software and Algorithms		
Prism v7.04	Graphpad	Prism v7.04
PANTHER (release 10/10/2018)	Mi et al., 2017	http://www.pantherdb.org
Flowjo v10.5.2	FlowJo, LLC	Flowjo v10.5.2
Proteome Discoverer v2.2	Thermo Fisher Scientific	Cat# OPTON-30808
Mascot v2.3	Matrix Science	http://www.matrixscience.com/server.html
R Studio v1.0.44	R Studio	https://www.rstudio.com
Jalview	Waterhouse et al., 2009	http://www.jalview.org
Biocoductor Packages (for LIMMA)	Huber et al., 2015	http://www.bioconductor.org
Other		
Vivacon 30kDa MWCO ultrafiltration units	Sartorius	VN01H22

CONTACT FOR REAGENT AND RESOURCE SHARING

Further information and requests for resources and reagents should be directed to and will be fulfilled by the Lead Contact, Edward J.D. Greenwood (ejdg2@cam.ac.uk).

EXPERIMENTAL MODEL AND SUBJECT DETAILS

Cell lines

CEM-T4 (female) T cells (AIDS Reagent Program, Division of AIDS, NIAD, NIH: Dr. JP Jacobs, (Foley et al., 1965) and J-LAT A1 (male) T cells (AIDS Reagent Program, Division of AIDS, NIAD, NIH: Dr E Verdin, (Jordan et al., 2003; Jordan et al., 2001) were cultured in RPMI supplemented with 10% FCS, 100 units/ml penicillin and 0.1 mg/ml streptomycin at 37°C in 5% CO2. HEK293T (female) embryonic kidney cells (Lehner laboratory stocks) were cultured in DMEM supplemented with 10% FCS, 100 units/ml penicillin and 0.1 mg/ml streptomycin at 37°C in 5% CO2. All cells were confirmed to be mycoplasma negative (MycoAlert, Lonza).

METHOD DETAILS

NL4-3 molecular clones

pNL4-3-dE-EGFP (derived from the HIV-1 molecular clone pNL4-3 but encoding Enhanced Green Fluorescent Protein (EGFP) in the *env* open reading frame (ORF), rendering Env non-functional) was obtained through the AIDS Reagent Program, Division of AIDS, NIAD, NIH: Drs Haili Zhang, Yan Zhou, and Robert Siliciano (Zhang et al., 2004) and the complete sequence verified by Sanger sequencing. The ΔVpr mutant was generated by cloning three stop codons into the Vpr open reading frame, immediately after the overlap with Vif to prevent interference with that gene:

WT Vpr ORF sequence

ATGGAACAAGCCCCAGAAGACCAAGGGCCACAGAGGGAGCCATACAATGAATGGACACTAGAGCTTTTAGAGGAA.

ΔVpr ORF sequence

ATGGAACAAGCCCCAGAAGACCAAGGGCCACAGAGGGAGCCATACAATGAATGGACACTAGAGCTTTAATAGTAA

Viral stocks

VSVg-pseudotyped NL4-3-dE-EGFP HIV viral stocks were generated by co-transfection with pMD.G (VSVg) as previously described (Greenwood et al., 2016). NL4-3-dE-EGFP HIV viral stocks were titerd by infection/transduction of known numbers of relevant target cells under standard experimental conditions followed by flow cytometry for GFP and CD4, using Anti-CD4-AF647 (clone OKT4; BioLegend) at 48 hr to identify % infected cells. Single Vpr and Vpx proteins were expressed in a modified dual promotor pHRSIN (van den Boomen et al., 2014) vector in which Vpr or Vpx expression is driven by the rous sarcoma virus (RSV) promotor, and Emerald GFP expression is driven by the ubiquitin promotor. Virus was generated by co-transfection with pCMVdr8.91 and pMD.G (Zufferey et al., 1997) in 293T as previously described (Greenwood et al., 2016). Infectious MOI was normalized by infection in the absence of reverse transcription (RTi) inhibitors, which were included where specified (see below). GenBank accession numbers of the untagged sequences for the Vpr and Vpx proteins used are given in the Key Resources Table. Amino acid sequences were codon optimized, modified to include a HA tag, and synthesized as double stranded DNA (IDT), inserted into the empty vector construct by Gibson assembly, and confirmed by sanger sequencing.

Lentivector for shRNA expression

For lentiviral DCAF1 shRNA-mediated knockdown, hairpins were cloned into pHRSIREN-PGK-hygro, with transduced cells selected for hygromycin resistance. The same methodology was used to clone other target shRNA haripins, into pHRSIREN-PGK-puro (encoding puromycin resistance). Gene specific target sequences were chosen from the Broad institute GPP web portal, and are provided in the forward orientation in the Key Resources Table.

CEM-T4 T cell infections

CEM-T4 T cells were infected with concentrated NL4-3-dE-EGFP or pHRSIN lentiviral stocks by spinoculation at 800 × g for 1 h in a non-refrigerated benchtop centrifuge in complete media supplemented with 10 mM HEPES. Where reverse transcription treatment was specified (RTi), cells were incubated with zidovudine (10 μM) and efavirenz (100 nM) (AIDS Reagent Program, Division of AIDS, NIAD, NIH) for 1 hr prior to spinoculation, and inhibitors maintained at these concentrations during subsequent cell culture. For MS experiments, cells were subject to dead cell removal (magnetic dead cell removal kit, Miltenyi). Subsequent sample preparation, TMT labeling, and MS are described below, at the end of this section.

Immunoblotting

Antibodies against the following proteins were used for immunoblot, listed by manufacturer: Atlas antibodies: TASOR (HPA006735). Bethyl: BBX (A303-151A), HLTF (A300-230A), RALY (A302-070A), ZNF512B (A303-234A). Cell Signaling Technology: SMN1/2 (2F1). Novus: ESCO2 (NB100-87021). Origene: UNG2 (2C12). Proteintech: Vpr (51143-I-AP). Santa Cruz: CCNB1 (SC-245), ZGPAT (SC-515524). Sigma: β-actin (AC74). Abcam: p24 (ab9071), VCP (ab11433). The following secondary antibodies were used: goat anti-mouse-HRP and goat anti-rabbit-HRP (immunoblot, Jackson ImmunoResearch, West Grove, PA). Blots were immersed in chemiluminescent substrate (SuperSignal West Pico or Dura, Thermo Fisher Scientific), and signal was visualized using X-ray film or the iBright CL1000 imaging system (Thermo Fisher Scientific).

ShRNA knockdown confirmation by RT-PCR

Total RNA was extracted using the RNeasy Plus Mini kit (QIAGEN). Total RNA (250 ng) was reverse transcribed into cDNA using a poly(d)T primers and SuperScript III Reverse Transcriptase (Invitrogen) following the manufacturer's instructions. Real-time qRT–PCR was performed using the ABI 7500 Real-Time PCR system (Applied Biosystems) and SYBR Green PCR Master Mix (Applied Biosystems), with cycling parameters of 50°C for 2 min and 95°C for 5 min, followed by 40 cycles of 95°C for 15 s and 60°C for 1 min. The gene-of-interest specific primer pairs were predesigned and purchased from Sigma-Aldrich as KiCqStart primers. The sequences are detailed in the key resources table. The difference in the amount of input cDNA was normalized to an internal control of GAPDH (Cano et al., 2015).

7-AAD staining

Cells were fixed in ice cold 70% ethanol for at least 30 minutes, washed with PBS and stained in 25ug/ml 7-AAD for 15 minutes before acquisition on a BD FACSCalibur. Analysis was carried out in Flowjo, v.10.5.2. Dead cells and doublets were excluded by gating on forward scatter, side scatter, and fluorescent area and height. Univariate cell cycle modeling was carried out using the Watson pragmatic method in Flowjo.

Immunoprecipitation Mass spectrometry (IP-MS)

Prior to infection with Vpr positive or negative lentivirus, CEM-T4 T cells were treated for two hours with the pan-cullin inhibitor MLN4924, 500nm (Millipore). 24 hours post infection, cells were lysed in 1% NP-40 (IGEPAL CA-630, Sigma-Aldrich), with benzonase (Sigma-Aldrich) at 2000 units/ml. Lysates were pre-cleared with IgG-Sepharose (GE Healthcare, UK) and incubated for 3 hr at 4°C with anti-HA coupled to agarose beads (EZview Red Anti-HA Affinity Gel, Sigma-Aldrich). After washing in 0.5% NP-40, samples were eluted with 0.5 mg/ml HA peptide (Sigma-Aldrich) at 37°C for 1 hr. Proteins defined as co-immunoprecipitating with Vpr were detected with at least 3 peptides in the Vpr condition, not identified in the control condition, and were present in < 20% of IP-MS available in the Crapome v1.1 database (Mellacheruvu et al., 2013).

Labeling with amino acids in cell culture (Pulsed-SILAC)

For SILAC labeling, CEM-T4 T cells were grown for at least 7 cell divisions in SILAC RPMI lacking lysine and arginine (Thermo Fisher Scientific) supplemented with 10% dialysed FCS (GIBCO, Thermo Fisher Scientific), 100 units/ml penicillin and 0.1 mg/ml streptomycin, 280 mg/L proline (Sigma, UK) and medium (K4, R6; Cambridge Isotope Laboratories, Tewksbury, MA) or heavy (K8, R10; Cambridge Isotope Laboratories) $^{13}C/^{15}N$-containing lysine (K) and arginine (R) at 50 mg/L. At 0 h, cells were washed in media containing only light (^{12}C /^{14}N) lysine and arginine and were maintained in this media for the duration of the experiment.

J-LAT reactivation experiments

JLAT A1 cells were transduced with Cre recombinase, NL4-3 Vpr or HIV-2 ROD Vpx within a pHRSIN IRES SBP-ΔLNGFR vector (Matheson et al., 2014). 24 h after transduction, cells were treated with 2 ng/ml TNFα (PeproTech, 300-01A). After an additional 24 h cells were stained with anti-CD271 (NGFR)-APC (clone ME20.4, Biolegend), and analyzed for NGFR and GFP expression by flow cytometry. Cells were gated for NGFR+ cells to exclude non-transduced cells. Flow cytometry data was acquired on a BD FACSCalibur.

Sample Preparation for Mass spectrometry

Samples were prepared using three different methods depending on the experiment. Initial infection experiment (Figure 1) was by SDC-FASP. Vpr particle (Figure 2) and shRNA (Figure S1) experiments were by PreOmics NHS-iST sample preparation Kit. pSILAC and IP-MS experiments were by SP3.

SDC-FASP

Samples prepared essentially according to the protocol in León et al. (2013). Briefly samples were lysed in 50mM TEAB (pH8.5) 2% SDS, reduced and alkylated with TCEP/Iodoacetamide, quantified by BCA assay. 50ug of each sample was diluted with TEAB/8M urea for loading onto 30kDa ultrafiltration devices. Samples were washed 3 times with 500uL urea buffer and 3 times with digestion buffer (TEAB/0.5% sodium deoxycholate) before resuspending in 50uL digestion buffer containing 1ug trypsin and incubating overnight at 37 degrees. After digestion samples were spun through the filters and filters washed with 50uL TEAB. SDC was removed by acidification and two phase partitioning with ethyl acetate before vacuum drying and labeling with TMT reagents according to the manufacturer's instructions.

PreOmics iST

Samples lysed in kit lysis buffer and quantified by BCA assay. 25ug of each sample was digested essentially according to manufacturer's instructions, scaling volumes for a digestion of 25ug total protein.

SP3

Samples were lysed in 50mM TEAB (pH8.5) 2% SDS (IP-MS were adjusted to 2% SDS), reduced and alkylated with TCEP/Iodoacetamide before digestion using the SP3 method (Hughes et al., 2014). Briefly, carboxylate modified paramagnetic beads are added to the sample and protein is bound to the beads by acidification with formic acid and addition of acetonitrile (ACN, final 50%). The beads are then washed sequentially with 100% ACN, 70% Ethanol (twice) and 100% ACN. 10-20uL TEAB (Triethylammonium bicarbonate)

pH8 and 0.1% Sodium deoxycholate (SDC) is then added to the washed beads along with trypsin. Samples were then incubated overnight at 37 degrees with periodic shaking at 2000rpm. After digestion, peptides are immobilised on beads by addition of 200-400uL ACN and washed twice with 100uL ACN before eluting in 19uL 2% DMSO and removing the eluted peptide from the beads.

Offline high pH reversed-phase (HpRP) peptide fractionation

For whole cell proteome samples HpRP fractionation was conducted on an Ultimate 3000 UHPLC system (Thermo Scientific) equipped with a 2.1 mm × 15 cm, 1.7μ Aqcuity BEH C18 column (Waters, UK). Solvent A was 3% ACN, Solvent B was 100% ACN, solvent C was 200 mM ammonium formate (pH 10). Throughout the analysis solvent C was kept at a constant 10%. The flow rate was 400 μL/min and UV was monitored at 280 nm. Samples were loaded in 90% A for 10 min before a gradient elution of 0%–10% B over 10 min (curve 3), 10%–34% B over 21 min (curve 5), 34%–50% B over 5 mins (curve 5) followed by a 10 min wash with 90% B. 15 s (100 μL) fractions were collected throughout the run. Peptide containing fractions were orthogonally recombined into 24 fractions (i.e., fractions 1, 25, 49, 73, 97 combined) and dried in a vacuum centrifuge. Fractions were stored at −80°C prior to analysis.

Mass spectrometry

Data were acquired on an Orbitrap Fusion mass spectrometer (Thermo Scientific) coupled to an Ultimate 3000 RSLC nano UHPLC (Thermo Scientific). HpRP fractions were resuspended in 20 μl 5% DMSO 0.5% TFA and 10uL injected. Fractions were loaded at 10 μl/min for 5 min on to an Acclaim PepMap C18 cartridge trap column (300 um × 5 mm, 5 um particle size) in 0.1% TFA. After loading a linear gradient of 3%–32% solvent B was used for sample separation over a column of the same stationary phase (75 μm × 50 cm, 2 μm particle size) before washing at 90% B and re-equilibration. Solvents were A: 0.1% FA and B:ACN/0.1% FA. 3h gradients were used for whole cell proteomics samples, 1h gradients for IP-MSs.

An SPS/MS3 acquisition was used for TMT experiments and was run as follows. MS1: Quadrupole isolation, 120’000 resolution, 5e5 AGC target, 50 ms maximum injection time, ions injected for all parallelisable time. MS2: Quadrupole isolation at an isolation width of m/z 0.7, CID fragmentation (NCE 35) with the ion trap scanning out in rapid mode from m/z 120, 8e3 AGC target, 70 ms maximum injection time, ions accumulated for all parallelisable time. In synchronous precursor selection mode the top 10 MS2 ions were selected for HCD fragmentation (65NCE) and scanned out in the orbitrap at 50’000 resolution with an AGC target of 2e4 and a maximum accumulation time of 120 ms, ions were not accumulated for all parallelisable time. The entire MS/MS/MS cycle had a target time of 3 s. Dynamic exclusion was set to ± 10 ppm for 90 s, MS2 fragmentation was trigged on precursor ions 5e3 counts and above. For IP-MS, MS2 instead used HCD fragmentation (NCE 34) and a maximum injection time of 250ms and had a target cycle time of 2 s. For pSILAC MS1 was acquired at 240’000 resolution.

QUANTIFICATION AND STATISTICAL ANALYSIS

Description of statistical tests and n values are provided in the figure legends. Anova and Fisher’s exact test analysis were carried out as described in figure legends using Graphpad Prism (v7.04).

Gene ontology enrichment

Gene ontology enrichment analysis was carried out using the statistical overrepresentation test of PANTHER (release 10/10/2018) (Mi et al., 2017), using the web interface at: http://www.pantherdb.org/, and using the GO Ontology database (release 06/09/2018), (Ashburner et al., 2000; The Gene Ontology Consortium, 2019). Lists of proteins highly significantly depleted or increased by Vpr were compared to a background list of proteins quantitated in the two experiments used to define those lists. p values shown are the results of a Fisher’s exact test with Bonferroni correction. In the case of GO: Cellular compartment analysis, this is highly conservative as it is corrected for all 1061 possible cellular compartment terms, not just those curated. When calculating the proportion of proteins with a specific GO term, only proteins with a GO term of that class were included in the denominator.

MS Data processing and analysis

For TMT labeled samples data were searched by Mascot within Proteome Discoverer 2.1 in two rounds of searching. First search was against the UniProt Human reference proteome (26/09/17) (UniProt Consortium, 2019), the HIV proteome and compendium of common contaminants (GPM). The second search took all unmatched spectra from the first search and searched against the human trEMBL database (Uniprot, 26/09/17). The following search parameters were used. MS1 Tol: 10 ppm, MS2 Tol: 0.6 Da. Enzyme: Trypsin (/P). MS3 spectra were used for reporter ion based quantitation with a most confident centroid tolerance of 20 ppm. PSM FDR was calculated using Mascot percolator and was controlled at 0.01% for ‘high’ confidence PSMs and 0.05% for ‘medium’ confidence PSMs. Normalization was automated and based on total s/n in each channel. Protein/peptide abundance was calculated and output in terms of ‘scaled’ values, where the total s/n across all reporter channels is calculated and a normalized contribution of each channel is output. Proteins/peptides satisfying at least a ‘medium’ FDR confidence were taken forth to statistical analysis in R (v3.3.1) (R Core Team, 2013). This consisted of a moderated t test (Limma) with Benjamini-Hochberg correction for multiple hypotheses to provide a q value for each comparison (Huber et al., 2015; Schwämmle et al., 2013). IP-MS were submitted to a similar search workflow with quantitative data being derived from MS1 spectra via proteome discover minora feature detector node. For pSILAC

experiments data were processed in MaxQuant and searched using Andromeda with similar search parameters (Cox and Mann, 2008). MaxQuant output was uploaded into Perseus for calculation of significance B (Tyanova et al., 2016). Where conditions were not carried out in triplicate, downstream analysis was limited to proteins identified with at least 3 unique peptides.

Phylogenetic tree of Vpr sequences

An existing nucleic acid sequence alignment of representative Vpr sequences from HIV-1, HIV-2 and SIVs from 22 primate species was downloaded from the Los Alamos National Laboratories HIV database (https://hiv.lanl.gov/content/sequence/NEWALIGN/align.html). A tree was generated using the percentage identity average distance in jalview (Waterhouse et al., 2009) and visualized in Figtree (http://tree.bio.ed.ac.uk/software/figtree). Sequences from 5 primate species not falling into the highlighted linages are not shown. *Cercopithecus* lineage includes closely related viruses from primates of the *Miopithecus* and *Piliocolobus* genera.

DATA AND SOFTWARE ANALYSIS

In addition to Table S1, which includes data from all of the proteomics experiments carried out here, proteomics data have been deposited to the ProteomeXchange Consortium via the PRIDE (Vizcaíno et al., 2016) partner repository. The accession number for the data reported in this paper is PRIDE: PXD013221.

Cell Reports, Volume *27*

Supplemental Information

Promiscuous Targeting of Cellular Proteins by Vpr Drives Systems-Level Proteomic Remodeling in HIV-1 Infection

Edward J.D. Greenwood, James C. Williamson, Agata Sienkiewicz, Adi Naamati, Nicholas J. Matheson, and Paul J. Lehner

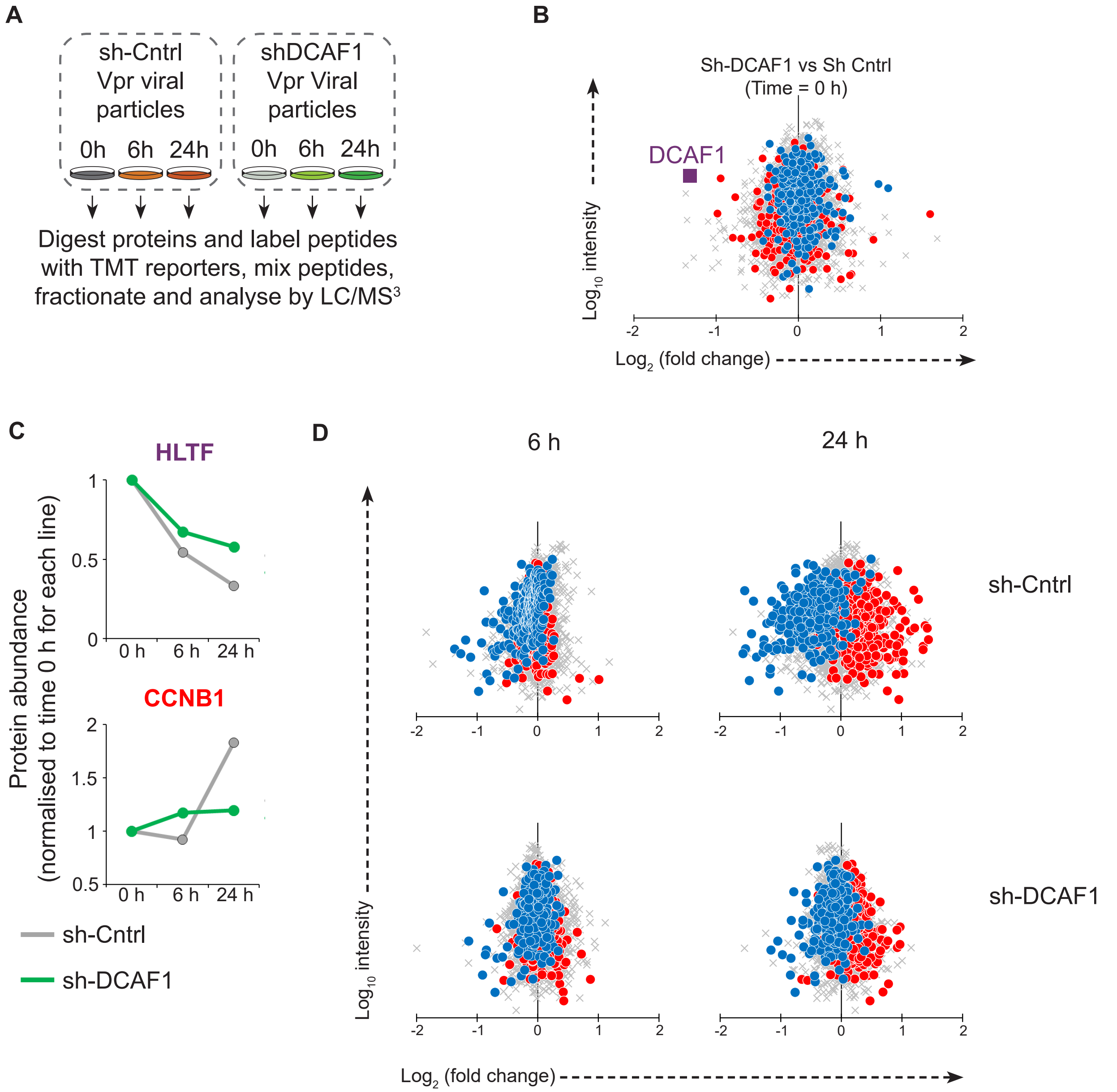

Figure S1. Quantifying the effect of Vpr under reduced DCAF1 conditions. Related to Figure 2.
A, Graphical summary of the DCAF1 KD experiment. **B**, Scatterplot displaying pairwise comparison between Sh-Control and Sh-DCAF1 cells at 0 h, with defined groups of 302 Vpr depleted (blue) and 413 increased (red) proteins highlighted. DCAF1 is highlighted in purple. **C**, Example time-course behaviour for one Vpr target (HLTF) and one secondary Vpr effect (CCNB1). **D**, Scatterplots showing the pairwise comparison between the 6 or 24 h time-point with the 0 h condition for each sh-transduced cell line.

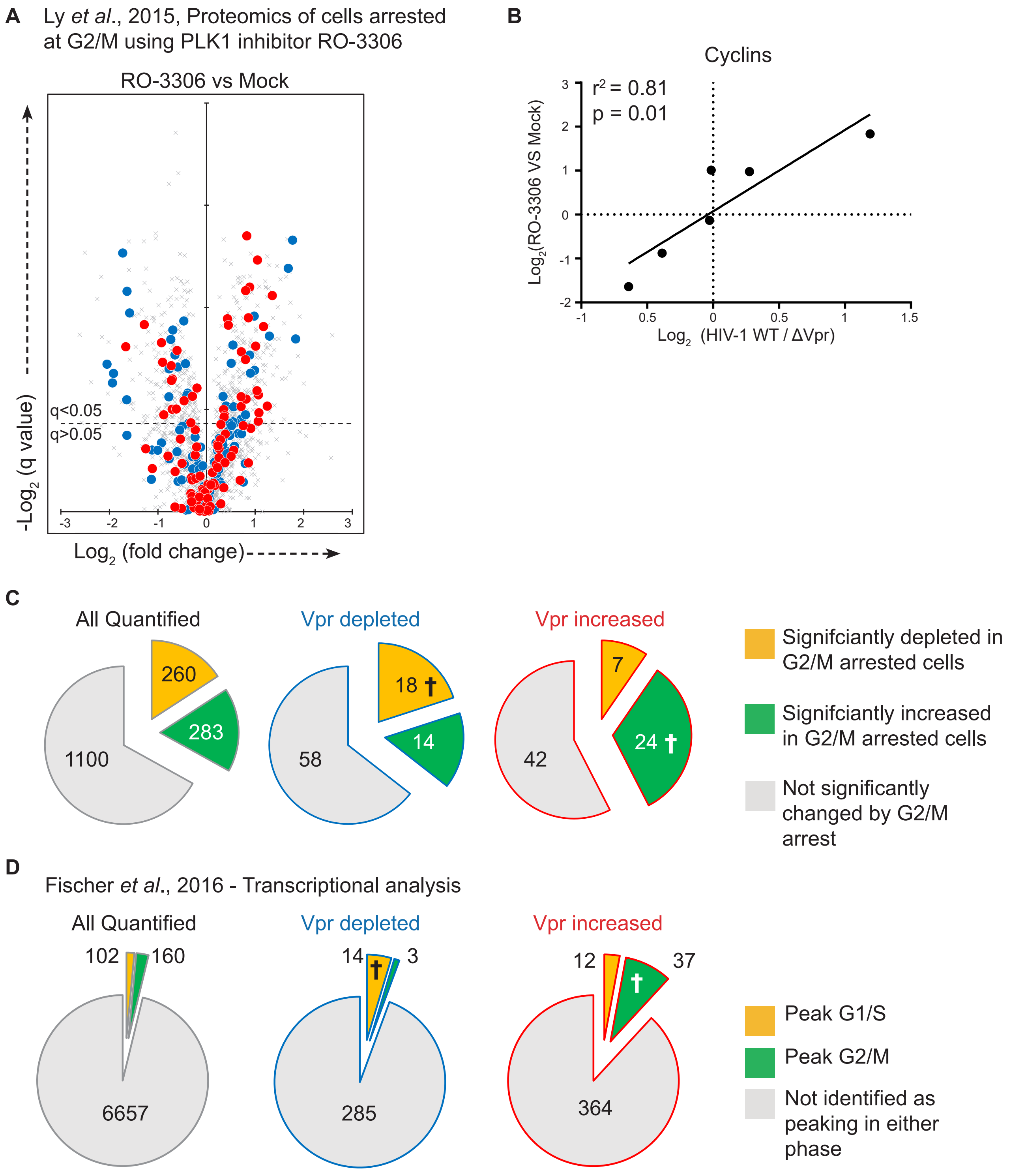
A
Ly et al., 2015, Proteomics of cells arrested at G2/M using PLK1 inhibitor RO-3306
RO-3306 vs Mock
q<0.05
q>0.05
-Log2 (q value)
Log2 (fold change)
-3 -2 -1 0 1 2 3
B
Cyclins
r2 = 0.81
p = 0.01
Log2(RO-3306 VS Mock)
Log2 (HIV-1 WT / ΔVpr)
3 2 1 0 -1 -2
-1 0.5 0 0.5 1 1.5
C
All Quantified
260
283
1100
Vpr depleted
18 †
14
58
Vpr increased
7
24 †
42
Signifciantly depleted in G2/M arrested cells
Signifciantly increased in G2/M arrested cells
Not significantly changed by G2/M arrest
D
Fischer et al., 2016 - Transcriptional analysis
All Quantified
102
160
6657
Vpr depleted
14
3
†
285
Vpr increased
12
37
†
364
Peak G1/S
Peak G2/M
Not identified as peaking in either phase

Figure S2. Cell cycle regulation of Vpr modulated proteins. Related to Figure 2.

A, Ly *et al*., 2015 contains a proteomic analysis of NB4 cells arrested in G2/M with the PLK1 inhibtor RO-3306. Scatterplot showing the pairwise comparison of abundance of proteins isolated from RO-3306 treated vs mock treated cells. Groups of proteins defined in the current study of 302 Vpr depleted (blue) and 413 increased (red) proteins are highlighted, indicating the behaviour of these proteins in NB4 cells arrested at G2/M. **B**, Correlation of the Vpr mediated change in cyclin abundance in the present study (x-axis), with RO-3306 mediated changes in NB4 cells in Ly *et al*., 2015. Changes in cyclin abundance in HIV infection are assumed to be secondary to cell cycle arrest, and thus this correlation indicates the concordance between effects secondary to cell cycle arrest between the two datasets. **C**, Pie charts showing the overlap between changes in the present study and changes induced by G2/M arrest in Ly *et al*., 2015. Left panel shows the behaviour of all proteins quantified in both the present study (Figure 1A and Figure 2A) and Ly *et al*., 2015, i.e. a total of 1643 proteins were quantified in both datasets, of which 1100 proteins did not significantly change in RO-3306 G2/M arrest, 260 proteins were significantly depleted in G2/M arrested cells, and 283 proteins were significantly increased in G2/M arrested cells. Middle panel shows the behaviour the defined group of 302 Vpr depleted proteins. I.e. of 302 proteins, a total of 90 proteins were detected in Ly *et al*., 2015, 58 of which did not significantly change in G2/M arrest, 18 were significantly depleted in G2/M arrest and 14 were significantly increased in G2/M arrest. Right panel shows the behaviour of the defined group of 413 Vpr increased proteins. † Indicates the fraction in each case where the change induced by cell cycle arrest is in the same direction as the effect of Vpr, i.e. the fraction for which cell cycle arrest could explain the Vpr mediated protein changes. **D**, Fischer *et al*., 2016, use a meta-analysis of transcriptional datasets to define lists of 115 proteins whose expression peaks in G1/S phase and 174 proteins whose expression peaks in G2/M phase. Pie-charts show the overlap between these lists and (left) all proteins detected in the present study, (middle) proteins defined as being depleted by Vpr, and (right) proteins defined as being increased by Vpr. As in the proteomics dataset, there is some enrichment of proteins with peak expression in G2/M within Vpr increased proteins, and some enrichment of proteins with peak expression in G1/S in Vpr depleted proteins, consistent with some effects being secondary to cell cycle arrest, but these effects are in the minority.

Table S2. Behaviour of detected previously described Vpr target proteins within these datasets. Related to Table 1.

Accession	Gene	Vpr necessary	Incoming Vpr sufficient	Degraded within 6 h	Co-IP	In direct target list (Table 1)
Q14527	HLTF	Yes	Yes	Yes	-	Yes
Q7L590	MCM10	Yes	Yes	ND[a]	-	No - protein not quantitated in in 6 hour pulsed SILAC or IP-MS
Q8N5A5	ZGPAT	Yes	Yes	Yes	-	Yes
Q96AY2	EME1	Yes	Yes	NS	-	No - depleted but with a Sig.B value of >0.01 in the pulsed SILAC experiment, not detected in IP
Q96NY9	MUS81	Yes	Yes	Yes	-	Yes
Q6N021	TET2	Yes	ND	ND	-	No - protein not quantitated in incoming Vpr experiment
P13051	UNG	Yes	Yes	NS	-	No - depleted but with a Sig.B value of >0.01 in the pulsed SILAC experiment. Detected in the IP-MS but with a single peptide

[a]ND – not detected or quantitated in this experiment.
